# Digitally Assisted Clinical Decision-Making in Traditional Chinese Medicine: Comparative Study of 5 Large Language Models

**DOI:** 10.2196/80167

**Published:** 2026-03-02

**Authors:** Weiwei Liu, Shuchang Miao, Qun Ma, Yuxin Li, Yinxiang Deng, Xiaoqiu Wang, Xinwei Zhang, Nuosha Ma, Hanchi Miao, Yang Si, Qingxia Shi, Lin Zhu, Hongtao Shang, Yue Wang

**Affiliations:** 1 Department of Preventive Medicine, Affiliated Hospital of Nanjing University of Chinese Medicine, Nanjing, China; 2 The First School of Clinical Medicine, Affiliated Hospital of Nanjing University of Chinese Medicine, Nanjing, China; 3 Affiliated Hospital of Nanjing University of Chinese Medicine, No. 155, Hanzhong Road, Qinhuai District, Nanjing, China, 86 13809023858; 4 Department of Rheumatology, Affiliated Hospital of Nanjing University of Chinese Medicine, Nanjing, China

**Keywords:** traditional Chinese medicine, large language models, clinical decision support, human-AI collaboration, digital health, artificial intelligence, medical informatics, clinical decision making

## Abstract

**Background:**

Traditional Chinese medicine (TCM) clinical decision-making involves complex integration of syndrome differentiation, constitutional assessment, and individualized treatment selection, creating challenges for standardization and quality assurance. While large language models (LLMs) demonstrate capabilities in medical knowledge integration and clinical reasoning, their application to TCM remains largely unexplored, particularly regarding syndrome differentiation principles and prescription formulation.

**Objective:**

This study evaluated 5 contemporary LLMs in TCM clinical decision-making and assessed human–artificial intelligence (AI) collaboration compared with independent approaches. Specific objectives were to benchmark LLM performance in TCM knowledge assessment, evaluate clinical case analysis capabilities, identify the optimal model, and assess the quality, efficiency, and acceptability of human-AI collaboration.

**Methods:**

In total, 5 mainstream LLMs were evaluated—Claude 3.7 Sonnet-Extended (Anthropic), ChatGPT 4.5 (OpenAI), Grok3-DeepSearch (xAI), Gemini 2.0 Flash Thinking Experimental (Google), and DeepSeek-R1 (DeepSeek). The evaluation consisted of four phases, (1) TCM knowledge assessment using 160 standardized questions, (2) clinical case analysis of 30 cases representing different disease systems and complexity levels, (3) optimal model selection using weighted scoring (40% knowledge and 60% clinical analysis), and (4) clinical application assessment involving 10 TCM practitioners and 2 experts comparing physician-only, AI-only, and human-AI collaboration across 5 clinical cases. Statistical analysis included descriptive statistics, reliability analysis, comparative testing, and regression analysis.

**Results:**

DeepSeek-R1 demonstrated superior performance across both evaluation domains, achieving 96.7% accuracy in knowledge assessment and 17.31/20 (SD 2.65) in clinical case analysis, significantly outperforming other models (*P*<.001). Human-AI collaboration achieved significant improvements compared with physician-only decision-making, with 16.1% quality enhancement (33.62 vs 28.97; *P*<.001) and 66.1% time reduction (162.6 s vs 479.2 s; *P*<.001). System usability was rated favorably (System Usability Scale score=76.8; *P*=.002), with high acceptance rates (74.25% adoption, 24% modification, and 1.75% rejection). AI assistance provided the greatest benefits in prescription formulation and medication selection (*P*<.001).

**Conclusions:**

LLMs, particularly DeepSeek-R1, demonstrate substantial capabilities in TCM knowledge assessment and clinical case analysis. Human-AI collaboration significantly enhanced clinical decision-making quality and efficiency while maintaining high physician acceptance. These findings provide compelling evidence for the clinical value of AI-assisted decision-making in TCM, suggesting potential solutions to current challenges in knowledge standardization, clinical training, and health care delivery efficiency. Strategic implementation of AI assistance could significantly enhance the quality, efficiency, and accessibility of TCM care while preserving fundamental principles of individualized treatment.

## Introduction

### Background and Rationale

Traditional Chinese medicine (TCM) clinical decision-making involves complex integration of syndrome differentiation, constitutional assessment, and individualized treatment selection. Unlike Western medicine’s disease-centered approach, TCM uses syndrome-pattern recognition (“bian zheng lun zhi” meaning treatment based on pattern differentiation) that synthesizes information from multiple diagnostic modalities to characterize the patient’s overall pathophysiological state [1]. Two patients with identical biomedical diagnoses may receive entirely different TCM treatments based on variations in syndrome patterns—for instance, chronic gastritis may present as liver-qi stagnation, spleen deficiency, or stomach-yin deficiency, each requiring distinct therapeutic approaches. This individualization, while therapeutically advantageous, creates challenges for standardization and quality assurance. Furthermore, TCM expertise relies heavily on tacit knowledge accumulated through clinical experience, creating bottlenecks in practitioner training and contributing to geographic disparities in care quality.

Large language models (LLMs) demonstrate remarkable capabilities in medical knowledge integration and clinical reasoning tasks, with recent models achieving performance approaching human physician levels on medical licensing examinations [2,3]. These capabilities suggest potential applications in addressing knowledge management challenges, supporting clinical decision-making, and augmenting practitioner capabilities. However, applying these technologies to TCM presents unique challenges. TCM syndrome differentiation requires pattern recognition according to theoretical frameworks, including yin-yang balance and 5-element correspondences—reasoning patterns that differ substantially from biomarker-driven diagnostic logic. Prescription formulation exemplifies this complexity—classical formulas use sovereign-minister-assistant-courier hierarchies with individualized modifications based on subtle clinical distinctions. Additionally, TCM terminology presents semantic challenges, as concepts like “liver-qi stagnation” represent theoretical constructs without direct biomedical equivalents.

### Previous Work and Research Gap

Existing artificial intelligence (AI) research in TCM has primarily focused on specific diagnostic tasks, such as tongue diagnosis and pulse analysis, addressing narrow functions rather than comprehensive clinical decision-making [4,5]. These systems implement computer vision or signal processing for isolated diagnostic components; yet, comprehensive evaluation of AI capabilities across the full spectrum of TCM clinical competencies—including syndrome differentiation and prescription formulation—remains absent.

AI applications in Western medicine have demonstrated broader clinical value, with studies showing improved diagnostic accuracy and efficiency through human-AI collaboration [6-8]. Recent randomized controlled trials provide compelling evidence that LLM assistance enhances physician clinical reasoning, with the greatest benefits in complex scenarios requiring the synthesis of multiple information sources [9]. The nature of human-AI interaction in clinical decision-making requires careful consideration of role allocation. AI systems excel at rapid information processing, comprehensive database access, and consistent rule application, while human clinicians contribute contextual judgment, patient-specific considerations, and ethical reasoning [10,11]. Effective collaboration positions AI as a decision support tool providing recommendations for physician review, rather than autonomous decision-makers—preserving clinical authority while leveraging AI capabilities. This framework proves particularly relevant for TCM, where clinical decisions must integrate standardized knowledge with individualized treatment principles.

Current gaps include the absence of comprehensive LLM benchmarking studies in TCM contexts and the lack of evidence regarding human-AI collaboration effectiveness in traditional medicine. Understanding which clinical domains benefit most from AI assistance and optimal collaboration strategies remains essential for clinical implementation.

### Study Objectives and Hypotheses

This study aimed to evaluate 5 contemporary LLMs in TCM clinical decision-making and assess human-AI collaboration effectiveness. Specific objectives were to (1) benchmark LLM performance in TCM knowledge assessment; (2) evaluate clinical case analysis capabilities; (3) identify the optimal performing model; and (4) assess the quality, efficiency, and acceptability of human-AI collaborative decision-making.

We hypothesized that significant performance variations would exist among LLMs, with models having enhanced access to Chinese medical literature demonstrating superior performance. We further hypothesized that human-AI collaboration would demonstrate superior clinical decision quality and efficiency compared with independent approaches, particularly in complex areas such as prescription formulation.

## Methods

### Selection and Configuration of LLM

Model selection was based on three criteria: (1) release timing within January-February 2025 to ensure contemporaneous comparison and avoid confounding temporal effects, (2) accessibility through official application programming interfaces or web interfaces for reproducible testing, and (3) Chinese language proficiency, essential for TCM terminology comprehension and syndrome differentiation reasoning.

This study selected 5 mainstream LLMs for evaluation, including Claude 3.7 Sonnet-Extended (Anthropic), ChatGPT 4.5 (OpenAI), Grok3-DeepSearch (xAI), Gemini 2.0 Flash Thinking Experimental (Google), and DeepSeek-R1 (DeepSeek), all of which were released between January and February 2025. Claude 3.7 Sonnet-Extended is built on an improved transformer architecture with enhanced reasoning capabilities; ChatGPT 4.5 optimized knowledge synthesis functions based on the GPT transformer framework; Grok3-DeepSearch integrates transformer models with real-time retrieval and reasoning mechanisms; Gemini 2.0 Flash Thinking Experimental embeds experimental reasoning modules within the Gemini transformer architecture, and DeepSeek-R1 uses a reinforcement learning–enhanced transformer architecture without supervised fine-tuning. All models were accessed through their official application programming interfaces or web interfaces under standard configuration environments, with all testing completed in March 2025. The testing process used uniform input formats to ensure fair comparison across all evaluation systems. Table 1 provides detailed characteristics and configuration information for all evaluated models.

**Table 1.** Large language model characteristics and configuration details^a^.

Model	Developer	Release date	Architecture	Key features for TCM^b^ applications	Clinical advantages
DeepSeek-R1	DeepSeek	January 20, 2025	Mixture-of-Experts Transformer (671B total, 37B activated; large-scale RL^c^ without SFT^d^)	Large-scale RL training, enhanced reasoning, open-source	Cost-efficient, performance comparable to leading models, no supervised fine-tuning
Claude 3.7 Sonnet-Extended	Anthropic	February 23, 2025	Transformer with extended thinking (hybrid reasoning; customizable thinking budget)	“Extended thinking” capability, hybrid reasoning model	Switches between rapid response and deep reflection, enhanced instruction following
Gemini 2.0 Flash Thinking Experimental	Google	February 4, 2025	Transformer with reasoning modules (transparent chain-of-thought; 1M token context)	Transparent reasoning process, mathematical reasoning	Fast response, reasoning transparency, complex problem-solving
ChatGPT 4.5	OpenAI	February 27, 2025	GPT Transformer framework (enhanced post training; reduced hallucination)	Improved accuracy, contextual understanding	Reduced hallucination, broad knowledge base, suitable for medical tasks
Grok3-DeepSearch	xAI	February 18, 2025	Transformer with integrated search (DeepSearch agent; trained on Colossus supercluster)	“DeepSearch” functionality, real-time information retrieval	Advanced reasoning, real-time search integration, extensive computational training

a All models were accessed through official application programming interfaces or web interfaces under standard configurations during March 2025 testing period.

b TCM: traditional Chinese medicine.

c RL: reinforcement learning.

d SFT: supervised fine-tuning.

These models represent diverse architectural paradigms and training methodologies. DeepSeek-R1 uses a mixture-of-experts architecture with 671 billion total parameters and 37 billion activated per forward pass, using large-scale reinforcement learning without supervised fine-tuning [12]. Claude 3.7 Sonnet-Extended is the first hybrid reasoning model that can produce quick responses or extended step-by-step thinking with customizable thinking budgets for complex reasoning tasks [13]. Gemini 2.0 Flash Thinking implements transparent chain-of-thought reasoning with extended context processing (1 million token context window) [14]. GPT-4.5 uses enhanced posttraining methodology to reduce hallucination and broaden knowledge base coverage [15]. Grok3-DeepSearch integrates real-time web search capabilities through its DeepSearch agent, trained on the Colossus supercluster [16]. These architectural differences enable comparative evaluation of distinct technical approaches to medical reasoning tasks.

### Evaluation Framework

The evaluation methodology of this study consists of 4 parts. The first part is TCM fundamental knowledge assessment, using 160 single-choice questions from the TCM Practitioner Qualification Examination (Multimedia Appendix 1), with sample size determined through effect size analysis (Cohen *d*=0.5, power=0.80, α=.05). Questions were selected through stratified sampling to ensure balanced representation across 8 TCM disease systems—pulmonary disorders (24/160, 15%), cardiac disorders (8/160, 5%), cerebral disorders (16/160, 10%), spleen-stomach disorders (24/160, 15%), hepatobiliary disorders (24/160, 15%), renal disorders (24/160, 15%), qi-blood-fluid disorders (24/160, 15%), and limb-meridian disorders (16/160, 10%). Difficulty stratification included basic-level questions (n=60, 37.5%), intermediate-level questions (n=60, 37.5%), and advanced-level questions (n=40, 25%), with difficulty classification jointly determined by 6 experienced TCM practitioners through consensus evaluation. Each model was independently tested using standardized input formats and triple repetition testing. The second part included 30 clinical cases from the TCM Practitioner Qualification Examination, selected through stratified sampling (Multimedia Appendix 2). Cases were distributed across 7 TCM disease systems—pulmonary (8/30, 26.7%), cardiac (6/30, 20%), spleen-stomach (5/30, 16.7%), hepatobiliary (5/30, 16.7%), qi-blood-fluid (3/30, 10%), renal (2/30, 6.7%), and limb-meridian (1/30, 3.3%) disorders. Demographic characteristics included sex distribution (20/30, 66.7% male and 10/30, 33.3% female) and age stratification (≤19 y: 1/30, 3.3%, 20-39 y: 7/30, 23.3%, 40-59 y: 13/30, 43.3%, and ≥60 y: 9/30, 30%). Complexity (15/30, 50% simple and 15/30, 50% complex) was determined by 6 TCM physicians, with simple cases defined as single-system pathology and complex cases as multisystem involvement. Clinical case analysis established 7 scoring dimensions based on TCM Practitioner Examination standards. Quality control measures included blinded evaluation processes, evaluator training and calibration meetings, and dual independent assessments.

The third part implemented weighted calculation (40/100, 40% fundamental knowledge, and 60/100, 60% clinical cases) to determine optimal model selection. This allocation reflects TCM competency frameworks emphasizing clinical skills as the most important practitioner competency [17,18]. Clinical case analysis requires complex syndrome differentiation and prescription formulation—core competencies aligned with AI-assisted decision-making. Sensitivity analyses testing alternative weighting schemes (30:70, 50:50, 60:40, and 70:30) assessed selection robustness. The fourth part used 5 previously reported real clinical cases to evaluate clinical application effectiveness and human-AI collaboration models (Multimedia Appendix 3). The study enrolled 10 TCM practitioners with more than 5 years of clinical experience and 2 chief physician-level experts, comparing the clinical performance of the physician group, AI group, and human-AI collaboration group, respectively. The specific method of human-AI collaboration involved LLMs first providing complete syndrome differentiation and treatment plans, which were reviewed and modified by physicians to form the final treatment plans. Throughout the process, decision-making time and situations of plan adoption, modification, or rejection were recorded.

### Outcome Measures

Primary outcome measures included fundamental theoretical knowledge test accuracy rates, comprehensive clinical case analysis scores, and quality differences between the human-AI collaboration mode and the independent decision-making mode. Secondary outcome measures covered decision-making time efficiency, clinical application value assessment, physician experience, and usability scores (Multimedia Appendix 4), and detailed situations of adoption, modification, or rejection of plans during the collaboration process (Multimedia Appendix 5).

### Statistical Analysis

Statistical analysis implemented the following methods, (1) descriptive statistics were used to calculate overall accuracy rates and their 95% CIs for each model, with stratified accuracy analysis for different characteristics; (2) reliability analysis included using intraclass correlation coefficient (ICC) to assess interrater consistency, Fleiss κ coefficient to assess intramodel consistency, and Cohen κ coefficient to assess intermodel consistency, comparative analysis used nonparametric testing methods such as Friedman test and Nemenyi post hoc test, paired comparisons used McNemar test, and interaction analysis used 2-factor ANOVA; regression analysis was conducted to determine factors predicting answer accuracy rates and calculate odds ratios (ORs) with their 95% CIs; effect size calculation used Cohen *d* values for intergroup comparisons to quantify the degree of differences between different treatment conditions (Multimedia Appendix 6).

### Ethical Considerations

This study did not require ethical review as it used publicly available examination questions and published clinical cases. Participating physicians provided informed consent, and no patient privacy issues were involved.

## Results

### Overview

This study evaluated 5 LLMs in TCM clinical reasoning and assessed their integration into clinical practice. DeepSeek-R1 demonstrated the strongest performance in both knowledge assessment and clinical case analysis. Physician-AI collaboration significantly improved clinical decision quality and efficiency compared with physicians or AI working independently. The AI-assisted approach was well-accepted by practitioners, who retained final decision authority while benefiting from AI-generated recommendations.

### Phase 1: TCM Knowledge Assessment Performance

#### Overall Model Performance

Across 160 TCM knowledge single-choice questions with 3 repetitions per question, totaling 480 responses per model, the 5 LLMs demonstrated significant performance differences. DeepSeek-R1 achieved the highest overall accuracy rate of 96.7%, significantly outperforming all other models. Claude 3.7 Sonnet-Extended ranked second with an accuracy rate of 66.3%, followed by Gemini 2.0 Flash Thinking Experimental with 65.6% accuracy. GPT-4.5 and Grok3-DeepSearch performed poorly, with accuracy rates of 52.5% and 50.6%, respectively. illustrates the comparative performance across all models, with Table 2 providing comprehensive accuracy metrics and reliability measures.

**Table 2.** Traditional Chinese medicine knowledge assessment performance across models^a^ (*P*<.001 for all based on the Friedman test for overall comparison across models).

Model	Overall accuracy, % (95% CI)	Basic level, % (95% CI)	Intermediate level, % (95% CI)	Advanced level, % (95% CI)	Internal consistency (Fleiss κ)
DeepSeek-R1	96.7 (94.7‐97.9)	100.0 (97.9‐100.0)	98.3 (95.2‐99.5)	89.2 (82.3‐93.6)	0.969
Claude 3.7	66.3 (61.9‐70.3)	85.6 (79.6‐90.0)	66.1 (58.9‐72.7)	37.5 (29.2‐46.6)	0.764
Gemini 2.0	65.6 (61.3‐69.7)	86.1 (80.2‐90.5)	62.2 (54.9‐69.1)	40.0 (31.5‐49.1)	0.953
GPT-4.5	52.5 (48.0‐56.9)	67.2 (60.0‐73.7)	47.2 (40.0‐54.6)	38.3 (30.0‐47.4)	0.803
Grok3	50.6 (46.2‐55.1)	67.2 (60.0‐73.7)	52.8 (45.4‐60.0)	22.5 (15.8‐30.9)	0.614

a Based on 160 single-choice questions from Traditional Chinese Medicine Practitioner Qualification Examination, with 3 repetitions per question (total 480 responses per model).

b Friedman test for overall comparison across models.

#### Disease System and Difficulty Level Analysis

**Figure 1.**
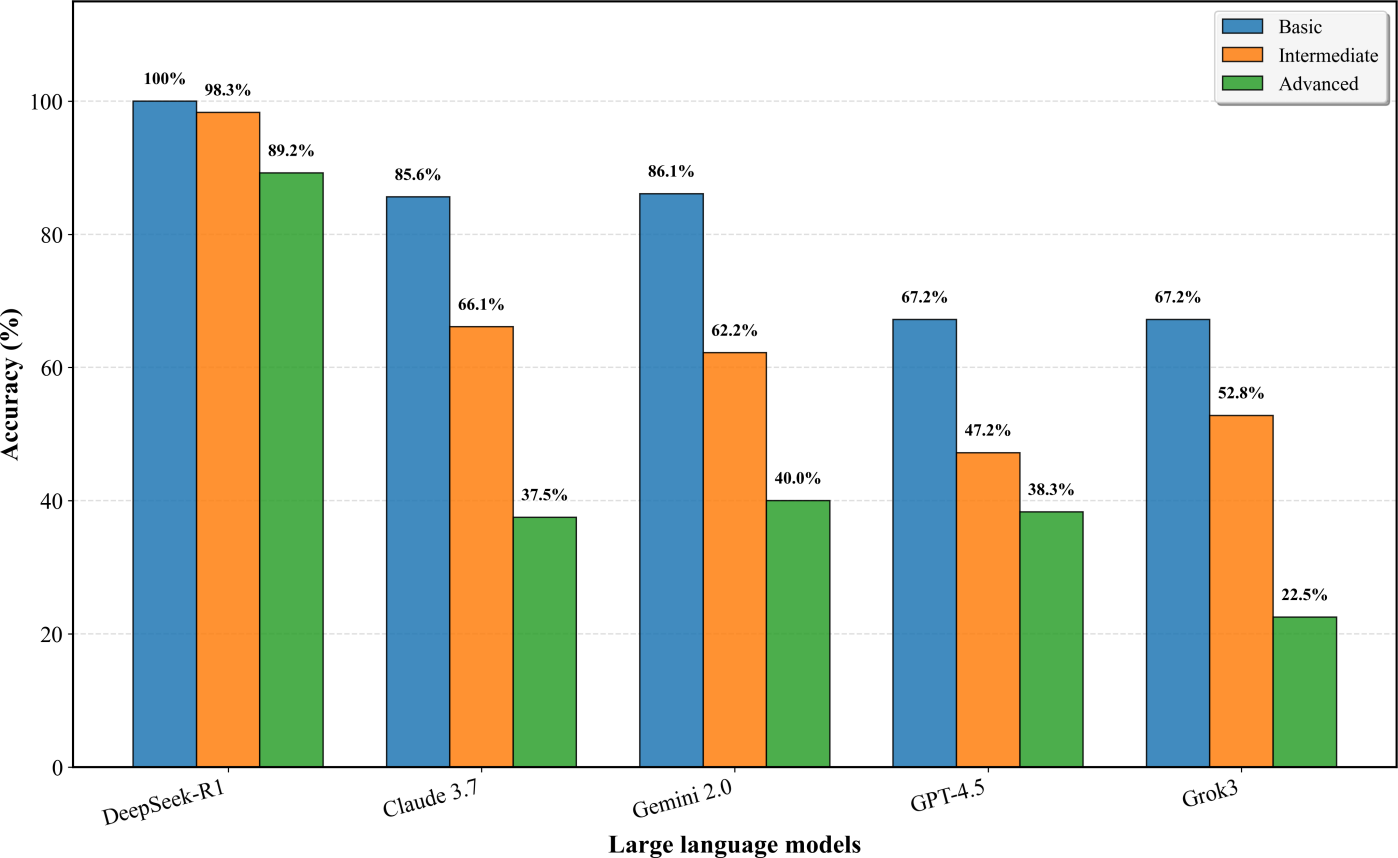
Model accuracy across 3 difficulty levels in traditional Chinese medicine knowledge evaluation.

In the evaluation across 8 major TCM disease systems, DeepSeek-R1 consistently performed optimally, with accuracy rates ranging from 91.7% (blood and fluid disorders) to 100% (pulmonary disorders). Claude 3.7 showed moderate but variable performance, achieving the highest accuracy of 87.5% in limb and meridian disorders and the lowest of 52.8% in blood and fluid disorders. Stratified analysis by question difficulty revealed a clear gradient effect across all models—basic-level questions generally achieved higher accuracy than advanced-level questions. DeepSeek-R1 maintained excellent performance across all difficulty levels with accuracy rates of 100% for basic questions, 98.3% for intermediate questions, and 89.2% for advanced questions, while other models showed a significant decline in advanced questions, such as Claude 3.7 decreasing from 85.6% to 37.5% and Gemini 2.0 from 86.1% to 40%. Figure 1 demonstrates the performance gradient across difficulty levels for all evaluated models.

#### Error Patterns and Model Consistency

Error pattern analysis indicated that “formula misuse” was the most common error type across all models, followed by “theory knowledge deficits” and “syndrome differentiation inaccuracy.” DeepSeek-R1 maintained the lowest error rates across all error categories (total error rate=3.3%), while other models had error rates ranging from 33.8% (Claude 3.7) to 49.4% (Grok3). Internal consistency analysis using Fleiss κ demonstrated excellent reliability for DeepSeek-R1 (κ=0.969) and Gemini 2.0 (κ=0.953), with moderate to good reliability for other models—Claude 3.7 (κ=0.764), GPT-4.5 (κ=0.803), and Grok3 (κ=0.614). Intermodal consistency analysis revealed moderate agreement between Claude 3.7 and Gemini 2.0 (Cohen κ=0.631), with generally lower consistency between other model pairs.

#### Statistical Comparisons and Predictive Factors

The Friedman test confirmed significant differences among models (*χ*²_₄_=159.2, *P<*.001). Nemenyi post hoc testing revealed significant differences between DeepSeek-R1 and all other models (all *P<*.001), as well as between Claude 3.7 and Grok3 (*P=*.04), and between Grok3 and Gemini 2.0 (*P=*.02). McNemar paired comparison tests indicated significant performance differences between most model pairs, with the largest effect observed between DeepSeek-R1 and other models. A 2-factor ANOVA showed significant main effects for both question difficulty (*F*_2,2385_*=*132.03; *P<*.001) and model type (*F*_4,2385_*=*93.43; *P<*.001), with a significant difficulty×model interaction (*F*_3,2385_*=*6.06; *P<*.001), indicating that performance gaps between models varied with question difficulty.

Logistic regression analysis performed well in predicting factors influencing correct responses (area under the curve=0.817, accuracy=74%). The analysis revealed that selecting the DeepSeek-R1 model was the strongest predictor of correct responses (OR 13.75, 95% CI 2.67‐2.80), followed by basic difficulty questions (OR 2.73, 95% CI 0.37‐0.39), and specific knowledge types including diagnostic key points (OR 2.68, 95% CI 2.60‐2.72) and treatment principles (OR 2.66, 95% CI 1.77‐1.85). Disease system analysis showed that compared with hepatobiliary disorders, limb and meridian disorders had higher odds of correct responses (OR 1.86, 95% CI 1.11‐1.16).

### Phase 2: Clinical Case Analysis Performance

#### Overall Case Analysis Scores

Analysis of 30 clinical cases (15 simple and 15 complex) using a 20-point scoring system revealed significant performance differences among models. DeepSeek-R1 achieved the highest overall mean score of 17.31 (SD 2.65; median 18.00, IQR 17.33-18.67), followed by Gemini 2.0 with a mean score of 16.16 (SD 3.06; median 17.00, IQR 15.00-18.58). Claude 3.7 demonstrated moderate performance with a mean score of 14.57 (SD 4.21; median 15.00, IQR 12.67-17.83), while Grok3 and GPT-4.5 showed lower performance with mean scores of 12.40 (SD 4.10; median 13.00, IQR 8.38-15.38) and 10.02 (SD 3.80; median 10.00, IQR 7.46-11.92), respectively. A 1-way ANOVA confirmed significant differences among models (*F*_4,145_=118.03; *P<*.001). Complete performance metrics across all evaluation dimensions are presented in Table 3.

**Table 3.** Clinical case analysis performance by model and evaluation dimensions^a^ (*P*<.001 based on one-way ANOVA for overall comparison across models).

Model	Overall score^b^, mean (SD)	Simple cases, mean (SD)	Complex cases, mean (SD)	Disease diagnosis^c^, mean (SD)	Syndrome diagnosis^c^, mean (SD)	Differentiation basis^d^, mean (SD)	Disease differentiation^c^, mean (SD)	Treatment method^e^, mean (SD)	Formula name^e^, mean (SD)	Medicinal composition^c^, mean (SD)
DeepSeek-R1	17.31 (2.65)	17.61 (2.26)	17.00 (2.96)	2.72 (0.85)	2.89 (0.41)	3.86 (0.44)	2.73 (0.58)	1.93 (0.25)	1.40 (0.68)	1.78 (0.71)
Gemini 2.0	16.16 (3.06)	16.53 (3.49)	15.78 (2.53)	2.49 (1.05)	2.80 (0.54)	3.79 (0.48)	2.59 (0.86)	1.83 (0.37)	1.24 (0.86)	1.41 (0.92)
Claude 3.7	14.57 (4.21)	15.19 (5.09)	13.96 (2.99)	2.33 (1.19)	2.68 (0.77)	3.51 (0.91)	2.42 (0.90)	1.81 (0.39)	0.84 (0.93)	0.99 (1.04)
Grok3	12.40 (4.10)	13.46 (3.73)	11.34 (4.20)	1.81 (1.20)	2.56 (0.85)	3.34 (1.16)	1.98 (1.14)	1.66 (0.53)	0.41 (0.67)	0.63 (0.91)
GPT-4.5	10.02 (3.80)	11.04 (3.80)	8.99 (3.53)	1.00 (1.22)	1.87 (1.26)	2.37 (1.26)	2.14 (0.98)	1.57 (0.67)	0.43 (0.72)	0.63 (0.87)

a Based on 30 clinical cases (15 simple and 15 complex) evaluated using Traditional Chinese Medicine Practitioner Examination standards. Values are mean (SD).

b Total score out of 20 points.

c Maximum score: 3 points.

d Maximum score: 4 points.

e Maximum score: 2 points.

#### Performance by Case Complexity

Complexity analysis revealed differential impacts across models. For complex cases, DeepSeek-R1 maintained the highest performance (mean 17.00, SD 2.96; median 18.00, IQR 17.00-18.67), followed by Gemini 2.0 (mean 15.78, SD 2.53; median 16.00, IQR 14.67-17.33), Claude 3.7 (mean 13.96, SD 2.99; median 15.00, IQR 12.83-15.00), Grok3 (mean 11.34, SD 4.20; median 13.00, IQR 8.08-14.75), and GPT-4.5 (mean 8.99, SD 3.53; median 10.00, IQR 7.08-11.17). For simple cases, the ranking remained similar with DeepSeek-R1 (mean 17.61, SD 2.26; median 18.00, IQR 17.67-18.83), Gemini 2.0 (mean 16.53, SD 3.49; median 18.00, IQR 15.33-18.83), Claude 3.7 (mean 15.19, SD 5.09; median 18.00, IQR 13.00-18.58), Grok3 (mean 13.46, SD 3.73; median 14.00, IQR 10.83-15.75), and GPT-4.5 (mean 11.04, SD 3.80; median 12.00, IQR 9.92-12.42). A 2-factor ANOVA revealed significant main effects for both model type (*F*_4,290_=122.62; *P<*.001) and case complexity (*F*_1,290_*=*32.75; *P<*.001), with no significant model×complexity interaction (*F*_4,290_*=*1.78; *P=*.13), indicating consistent performance patterns across complexity levels. The performance gap between simple and complex cases varied among models, with GPT-4.5 and Grok3 showing the largest decrements (2.06 and 2.11 points, respectively), while DeepSeek-R1 demonstrated the smallest impact (0.61 points).

#### Performance by Evaluation Dimensions

**Figure 2.**
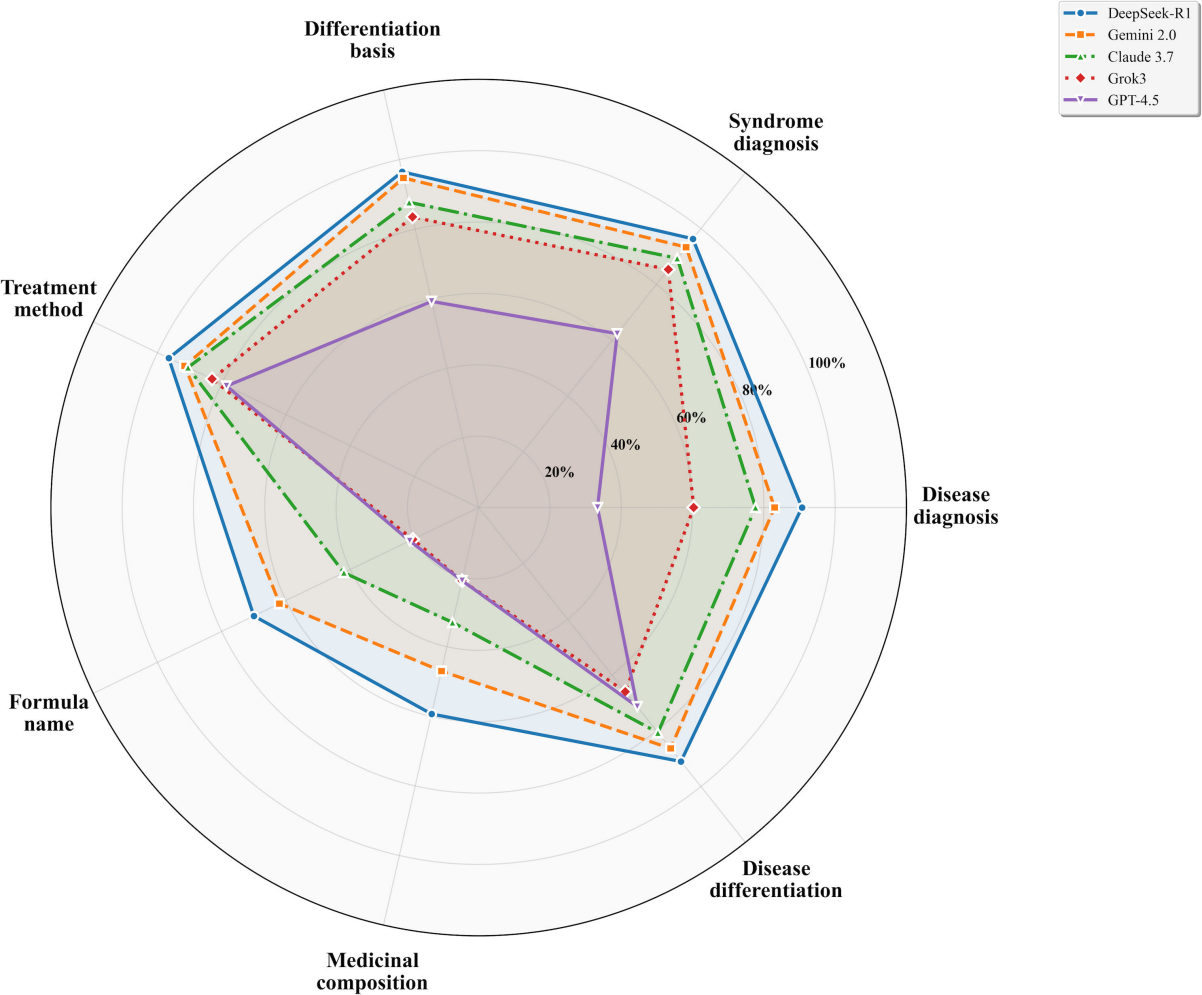
Clinical case analysis performance by evaluation dimensions for five large language models.

Dimensional analysis across seven evaluation criteria showed significant differences among models in all dimensions (all *P<*.001). DeepSeek-R1 consistently achieved the highest scores across dimensions—disease diagnosis (mean 2.72, SD 0.85), syndrome diagnosis (mean 2.89, SD 0.41), differentiation basis (mean 3.86, SD 0.44), treatment method (mean 1.93, SD 0.25), formula name (mean 1.40, SD 0.68), medicinal composition (mean 1.78, SD 0.71), and identification (mean 2.73, SD 0.58). Gemini 2.0 demonstrated competitive performance in most dimensions, particularly in differentiation basis (mean 3.79, SD 0.48) and syndrome diagnosis (mean 2.80, SD 0.54). Claude 3.7 showed moderate performance across all dimensions, with notable strengths in differentiation basis (mean 3.51, SD 0.91) and syndrome diagnosis (mean 2.68, SD 0.77). GPT-4.5 and Grok3 exhibited substantial deficits in formula name (GPT-4.5: mean 0.43, SD 0.72, and Grok3: mean 0.41, SD 0.67) and medicinal composition (GPT-4.5: mean 0.63, SD 0.87, and Grok3: mean 0.63, SD 0.91) dimensions, indicating particular challenges in prescription-related assessments. Figure 2 provides a visual comparison of model performance across all 7 evaluation dimensions using a radar chart representation.

#### Interrater Reliability and Model Stability

Interrater consistency analysis between evaluators demonstrated excellent agreement across all models. Gemini 2.0 showed perfect interrater agreement (mean absolute difference 0.00), followed by DeepSeek-R1 (0.033), Claude 3.7 (0.078), Grok3 (0.133), and GPT-4.5 (0.144). Model output stability analysis across multiple attempts revealed varying degrees of consistency, with Gemini 2.0 demonstrating the highest stability (mean 16.16, SD 0.55), followed by DeepSeek-R1 (mean 17.31, SD 0.69), Claude 3.7 (mean 14.57, SD 1.13), GPT-4.5 (mean 10.02, SD 1.99), and Grok3 (mean 12.40, SD 2.04). The stability analysis indicated that superior-performing models generally exhibited greater consistency in their outputs, with the exception of Gemini 2.0, which showed the highest stability despite moderate overall performance.

### Phase 3: Optimal Model Selection Results

Comprehensive scores were calculated using a weighted methodology with fundamental knowledge performance (40%) and clinical case analysis capability (60%). DeepSeek-R1 achieved the highest comprehensive score of 90.61 points, followed by Gemini 2.0 (74.72 points), Claude 3.7 (70.23 points), Grok3 (57.44 points), and GPT-4.5 (51.06 points). Based on superior performance across both evaluation domains and a significant advantage of 15.89 points over the second-ranked model, DeepSeek-R1 was selected as the optimal model for phase 4 clinical application and human-AI collaboration assessment.

To assess the robustness of the 40:60 weighting scheme, alternative knowledge:clinical ratios were tested (30:70, 50:50, 60:40, and 70:30). DeepSeek-R1 maintained first rank across all tested scenarios, with composite scores ranging from 89.59 to 93.63. The model demonstrated consistent superiority with margins of 14.87 to 23.45 points over the second-ranked model across all weighting variations. This finding confirms that the final model selection was robust to weighting parameter variations and would remain unchanged even with substantially different weighting schemes.

### Phase 4: Clinical Application and Human-AI Collaboration

#### Quality Comparison Analysis

Analysis of 5 clinical cases across 3 evaluation groups revealed significant performance differences. The AI-only group achieved the highest mean score of 33.80 (SD 1.12), followed closely by the human-AI collaboration group with 33.62 (SD 2.50), while the physician-only group scored 28.97 (SD 4.94). Kruskal-Wallis test confirmed significant differences among groups (H=35.003; *P<*.001). Pairwise comparisons revealed that human-AI collaboration significantly outperformed physician-only decision-making (paired *t*_49_=6.603, Cohen *d*=0.943; *P<*.001), representing a 116.1% quality improvement. No significant difference was observed between AI-only and human-AI collaboration groups (*t*_49_=−0.271, Cohen *d*=−0.074; *P=*.79), while AI-only significantly outperformed physician-only approaches (*t*_49_=−5.358, Cohen *d*=−1.015; *P<*.001). Comprehensive comparison results across all outcome measures are detailed in Table 4.

**Table 4.** Comparison of decision-making approaches in clinical case analysis^a^.

Outcome measure	Physician-only (n=50), mean (SD)	AI^b^-only (n=5), mean (SD)	Human-AI collaboration (n=50), mean (SD)	Statistical test	*P* value	Effect sizes^c^
Quality measures						
Total score	28.97 (4.94)	33.80 (1.12)	33.62 (2.50)	35.003^d^	<.001	0.943^e^
Disease diagnosis	4.41 (0.76)	4.40 (0.82)	4.51 (0.69)	0.474^d^	.79	No significant differences
Syndrome and treatment	3.65 (1.26)	4.55 (0.51)	4.31 (0.84)	7.510^d^	.02	Moderate to large effects
Prescription and medication	2.75 (0.78)	3.45 (0.37)	3.51 (0.57)	23.967^d^	<.001	Large effects favoring AI approaches
Precautions	2.39 (1.33)	4.00 (0.00)	4.00 (0.00)	76.680^d^	<.001	Very large effects favoring AI approaches
Efficiency measures						
Completion time (s)	479.2 (169.5)	86.0 (15.2)	162.6 (35.2)	14.541 (49)^f^	<.001	Large time reduction effects
Quality-time efficiency^g^	6.92 (3.19)	40.29 (7.00)	21.60 (4.74)	—^h^	—	Substantial efficiency improvements

a Based on evaluation of 5 clinical cases representing diverse disease systems and complexity levels.

b AI: artificial intelligence.

c Cohen *d* interpretation: small (0.2), medium (0.5), and large (0.8). Effect sizes reported for the primary comparison (human–artificial intelligence collaboration vs physician-only).

d Kruskal-Wallis test.

e Large positive effect: human–artificial intelligence collaboration significantly outperformed physician-only decision-making. Additional pairwise comparisons (physician-only vs artificial intelligence–only: Cohen *d*=−1.015; human–artificial intelligence collaboration vs artificial intelligence–only: Cohen *d*=−0.074) available upon request.

f Paired *t*-test for completion time comparison (human-artificial intelligence collaboration vs physician-only).

g Calculated as expert score/completion time×100.

h Not applicable.

#### Efficiency Analysis

**Figure 3.**
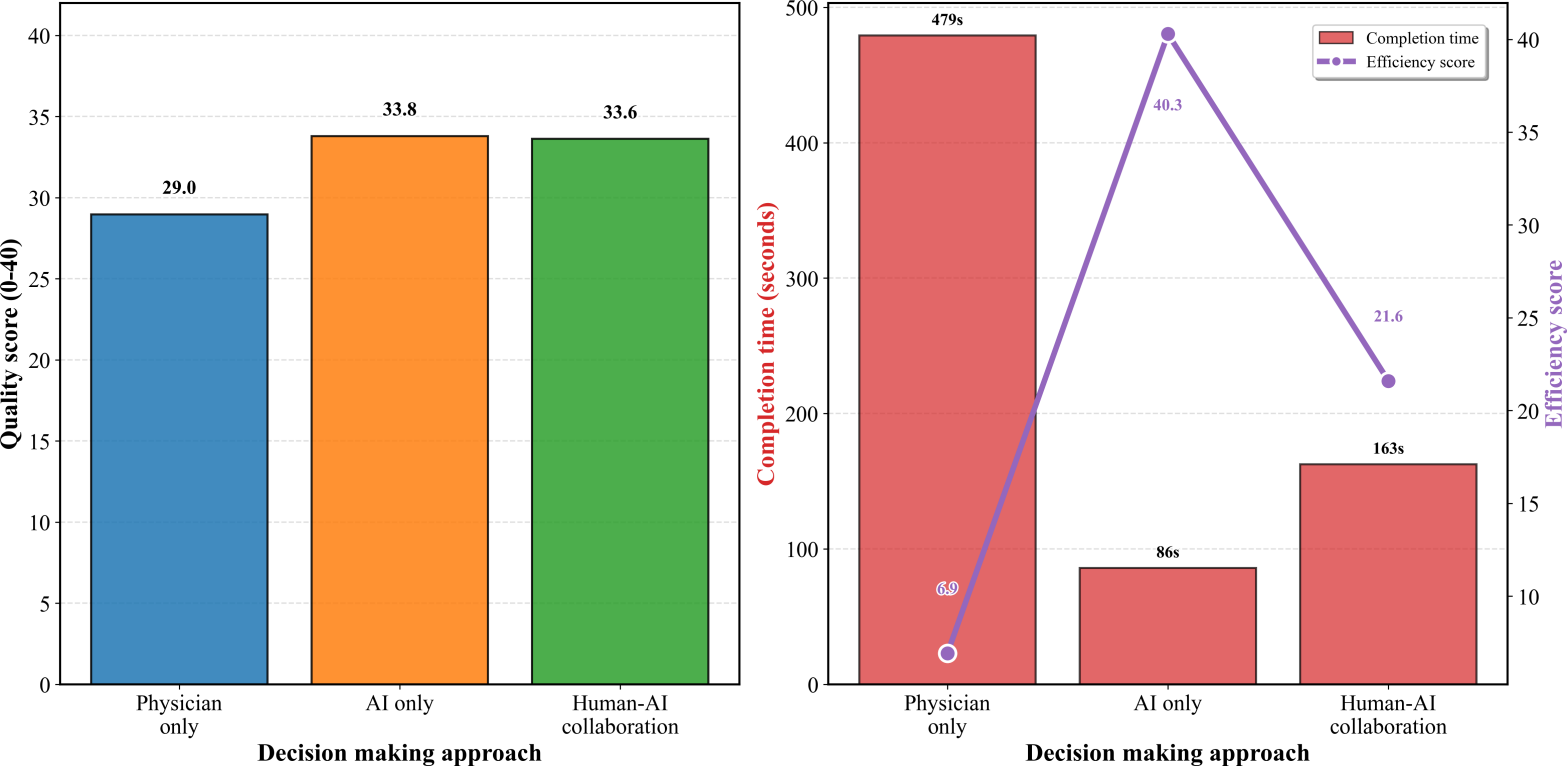
Quality and efficiency comparison across three decision-making approaches. AI: artificial intelligence.

Completion time analysis demonstrated substantial efficiency gains through human-AI collaboration. Physician-only decision-making required the longest time (mean 479.2, SD 169.5 s, range 160‐900 s), while AI-only analysis was fastest (mean 86.0, SD 15.2 s, range 70‐110 s). Human-AI collaboration achieved intermediate completion times (mean 162.6, SD 35.2 s, range 110‐270 s), representing a 66.1% time reduction compared with physician-only approaches. Paired *t* test confirmed significant time savings (*t*_49_=14.541; *P<*.001). Quality-time composite efficiency analysis (expert score/completion time×100) showed dramatic improvements—physician-only (mean 6.92, SD 3.19), human-AI collaboration (mean 21.60, SD 4.74), and AI-only (mean 40.29, SD 7.00), with human-AI collaboration demonstrating 212.2% efficiency improvement over physician-only decision-making. Figure 3 illustrates both quality improvements and efficiency gains achieved through human-AI collaboration.

#### Dimensional Performance Analysis

Evaluation across 5 core dimensions revealed varying collaboration benefits. Safety considerations showed consistently high performance across all groups with no significant differences (H=0.510; *P=*.78). Disease diagnosis demonstrated equivalent performance among groups (H=0.474; *P=*.79). However, significant improvements were observed in syndrome differentiation and treatment planning (H=7.510; *P=*.02), where human-AI collaboration (mean 4.31, SD 0.84) and AI-only (mean 4.55, SD 0.51) outperformed physician-only approaches (mean 3.65, SD 1.26). The most substantial benefits occurred in prescription and medication domains (H=23.967; *P<*.001), with human-AI collaboration (mean 3.51, SD 0.57) and AI-only (mean 3.45, SD 0.37) significantly exceeding physician-only performance (mean 2.75, SD 0.78). Precautions showed the largest effect size (H=76.680; *P*<.001), with both AI-only and human-AI collaboration groups achieving maximum scores (4.00) compared with physician-only approaches (mean 2.39, SD 1.33).

#### Expert Interrater Reliability

Interrater expert consistency analysis demonstrated excellent reliability across all evaluation dimensions. ICC revealed perfect agreement for Western diagnosis (ICC=1.000) and safety considerations (ICC=1.000), with near-perfect agreement for precautions (ICC=0.997, 95% CI 1.000‐1.000) and total scores (ICC=0.987, 95% CI 0.980‐0.990). Other dimensions showed strong to excellent reliability; TCM diagnosis (ICC=0.990, 95% CI 0.990‐0.990), treatment methods (ICC=0.986, 95% CI 0.980‐0.990), TCM syndrome differentiation (ICC=0.956, 95% CI 0.930‐0.970), medication composition (ICC=0.945, 95% CI 0.920‐0.960), and prescription formulation (ICC=0.861, 95% CI 0.800‐0.900). These findings confirmed the robustness and consistency of expert evaluations.

#### Clinical Application Value and Physician Experience

Clinical application value assessment revealed positive ratings across 3 domains. Clinical practicality achieved good ratings with operational feasibility (mean 3.60, SD 0.80), resource accessibility (mean 4.00, SD 0.77), and complex case analysis capability (mean 3.90, SD 0.83). Integration of TCM with Western medicine showed moderate ratings for laboratory interpretation (mean 3.30, SD 0.78), diagnostic accuracy (mean 3.10, SD 0.30), and evidence-based support (mean 3.30, SD 0.46). Clinical efficiency improvements were rated positively for diagnostic time impact (mean 4.10, SD 0.83), quality-time balance (mean 3.10, SD 0.83), and workload changes (mean 3.40, SD 1.11).

Physician experience evaluation demonstrated favorable collaboration outcomes. System usability received high ratings (mean 4.50, SD 0.67), while workflow integration showed moderate acceptance (mean 3.30, SD 0.64), and information presentation clarity was rated positively (mean 4.00, SD 0.77). Capability enhancement perception indicated moderate benefits for learning value (mean 3.20, SD 0.40), rare syndrome assistance (mean 3.20, SD 0.40), and clinical thinking expansion (mean 3.60, SD 0.80). Human-AI collaboration quality showed strong ratings for complementarity (mean 4.00, SD 0.77), workflow smoothness (mean 3.50, SD 0.67), and decision confidence enhancement (mean 3.90, SD 0.83).

#### System Usability and Collaboration Pattern Analysis

System Usability Scale evaluation yielded positive results with a mean score of 76.8 (SD 5.9, range 65.0‐87.5). A 1-sample *t* test confirmed that usability significantly exceeded the acceptable threshold of 68 points (*t*_9_=4.433; *P*=.002). Distribution analysis showed 10% (n=1) of physicians rated the system as excellent (≥80.3), 80% (n=8) as good (68‐80.3), and 10% (n=1) as needing improvement (<68).

**Figure 4.**
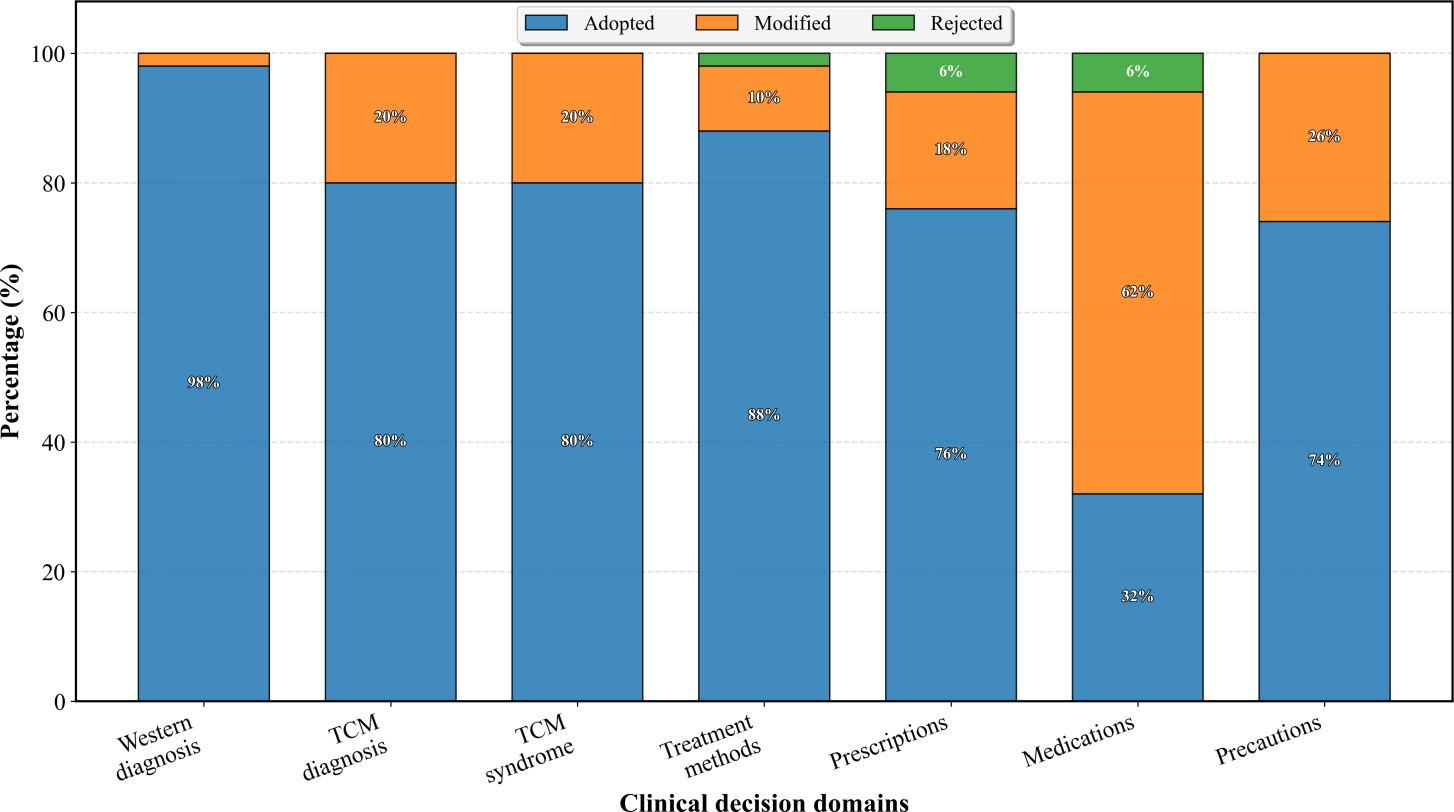
Human-AI collaboration patterns by clinical decision domains. AI: artificial intelligence; TCM: traditional Chinese medicine.

Collaboration pattern analysis across 400 total decisions revealed high acceptance rates—74.25% (297/400) adoption, 24% (96/400) modification, and 1.75% (7/400) rejection. Field-specific analysis showed variable adoption patterns; Western diagnosis achieved the highest adoption (98%), followed by TCM treatment methods (88%) and syndrome differentiation (80%), while medication recommendations showed lower adoption (32%) but high modification rates (62%). Common modification types included quality optimization (17 instances), medication adjustments (14 instances), and evidence supplementation (8 instances). Rejections were primarily due to philosophical differences (7 instances), predominantly in treatment philosophy and prescription selection domains. Figure 4 illustrates the detailed collaboration patterns across different clinical domains, while Table 5 presents comprehensive usability assessment and physician experience evaluation results.

**Table 5.** System usability assessment and physician experience evaluation^a^.

Assessment domain	Mean (SD)	Range	Rating level^b^
System Usability Scale			
Overall score^c^	76.8 (5.9)	65.0‐87.5	Good
Excellent (≥80.3), n (%)	—^d^	—	1 (10.0)
Good (68‐80.3), n (%)	—	—	8 (80.0)
Needs improvement (<68), n (%)	—	—	1 (10.0)
Clinical application value			
Clinical practicality			
Operational feasibility	3.60 (0.80)	2.0‐5.0	Good
Resource accessibility	4.00 (0.77)	3.0‐5.0	Good
Complex case analysis capability	3.90 (0.83)	2.0‐5.0	Good
TCM^e^-Western Medicine Integration			
Laboratory interpretation rationality	3.30 (0.78)	2.0‐5.0	Moderate
Diagnostic transformation accuracy	3.10 (0.30)	2.5‐3.5	Moderate
Evidence-based medicine support	3.30 (0.46)	2.5‐4.0	Moderate
Clinical efficiency enhancement			
Diagnostic time impact	4.10 (0.83)	2.0‐5.0	Good
Quality-time balance	3.10 (0.83)	2.0‐4.0	Moderate
Workload changes	3.40 (1.11)	1.0‐5.0	Moderate
Physician experience assessment			
Collaboration experience			
System usability	4.50 (0.67)	3.0‐5.0	Excellent
Workflow integration	3.30 (0.64)	2.0‐4.0	Moderate
Information presentation clarity	4.00 (0.77)	3.0‐5.0	Good
Capability enhancement perception			
Learning value	3.20 (0.40)	2.5‐4.0	Moderate
Rare syndrome assistance value	3.20 (0.40)	2.5‐4.0	Moderate
Clinical thinking expansion	3.60 (0.80)	2.0‐5.0	Good
Human-AI collaboration quality			
Human-AI^f^ complementarity	4.00 (0.77)	3.0‐5.0	Good
Collaboration smoothness	3.50 (0.67)	2.0‐4.0	Good
Decision confidence enhancement	3.90 (0.83)	2.0‐5.0	Good

a Based on responses from 10 traditional Chinese medicine practitioners with ≥5 years of clinical experience.

b Rating levels: 1.0‐2.4 (poor), 2.5‐3.4 (moderate), 3.5‐4.4 (good), and 4.5‐5.0 (excellent).

c One-sample *t*-test versus baseline score of 68: *t*=4.433, *P*=.002.

d Not applicable.

e TCM: traditional Chinese medicine.

f AI: artificial intelligence.

## Discussion

### Principal Results

This evaluation of 5 LLMs in TCM yielded significant findings. DeepSeek-R1 demonstrated superior performance in both knowledge assessment (96.7% accuracy) and clinical case analysis (mean score 17.31/20, SD 2.65). Human-AI collaboration enhanced decision-making quality by 16.1% while reducing time by 66.1%, with high system usability (System Usability Scale score=76.8) and acceptance rates (74.25% adoption). AI assistance provided the greatest benefits in prescription formulation and medication selection.

### Performance Analysis of LLMs

Substantial performance variations among models highlight the importance of systematic assessment in medical AI applications. DeepSeek-R1’s exceptional performance (96.7% accuracy) may stem from advantages in accessing Chinese medical literature and the domestic development context. The model’s consistent superiority across disease systems and difficulty levels indicates robust knowledge representation.

Error pattern analysis revealed formula misuse as the most common failure mode, reflecting the complexity of TCM prescription formulation. This underscores the challenge of integrating syndrome differentiation, constitutional assessment, and individualized treatment principles. DeepSeek-R1’s low error rates (3.3% total) suggest that leveraging extensive Chinese medical literature repositories through advanced natural language processing may be more effective than relying solely on general medical training datasets.

### Human-AI Collaboration Effectiveness

The demonstrated effectiveness of human-AI collaboration validates the complementary strengths of artificial and human intelligence in TCM decision-making. The 16.1% quality improvement achieved through collaboration represents a clinically meaningful enhancement, particularly given the already high baseline performance of experienced practitioners. The finding that AI-only and human-AI collaboration achieved comparable quality scores (mean 33.80, SD 2.50 vs mean 33.62, SD 1.12) while human-AI collaboration maintained superior consistency suggests that human oversight provides valuable quality assurance without compromising AI capabilities.

The efficiency gains observed (66.1% time reduction and 212.2% composite efficiency improvement) have profound implications for clinical practice scalability. The collaboration model appears to capture the rapid analytical capabilities of AI while preserving the contextual judgment and patient-specific considerations that characterize expert clinical practice. The dimensional analysis revealing selective benefits in prescription and medication domains suggests that AI assistance may be most valuable in knowledge-intensive tasks where comprehensive database access and pattern recognition provide clear advantages over individual practitioner knowledge.

### Clinical Implications

The findings have substantial implications for TCM practice transformation. The demonstration that AI assistance can significantly enhance both quality and efficiency suggests potential solutions to current health care challenges, including practitioner shortage, training standardization, and knowledge dissemination. The high adoption rates observed (74.25%) indicate that integration of AI assistance into existing clinical workflows may be feasible with appropriate system design and training protocols.

The educational implications are particularly noteworthy, as the AI system demonstrated consistent performance across complex cases that typically require years of clinical experience to master. This suggests potential applications in medical education, continuing professional development, and clinical decision support for junior practitioners. The detailed feedback and reasoning provided by AI systems may serve as valuable teaching tools, particularly in syndrome differentiation and prescription formulation, where tacit knowledge transfer has traditionally been challenging.

The findings also support the evolution toward precision medicine in TCM practice. The AI system’s ability to process comprehensive patient information and provide evidence-based recommendations may facilitate more individualized treatment approaches while maintaining consistency with TCM principles. This represents a significant advancement toward integrating traditional medical wisdom with contemporary health care delivery models.

### Comparison With Previous Work

This study provides the first comprehensive evaluation of LLMs in TCM applications. While previous studies demonstrated AI effectiveness in Western medicine diagnostic tasks [6-8], and recent randomized trials show that LLM assistance enhances physician clinical reasoning [9], TCM-specific challenges—including syndrome differentiation and individualized treatment formulation—remained largely unexplored. Our findings extend the demonstrated benefits of human-AI collaboration to traditional medicine contexts [10,11].

The high modification rates in medication domains (62%) contrast with high adoption rates in other medical AI applications [19], reflecting TCM practitioners’ preferences for individualized prescription modifications. This underscores the importance of AI systems that support rather than replace clinical judgment.

### Framework of Human-AI Collaboration

It is essential to clarify that the AI systems evaluated in this study function as clinical-decision support tools rather than autonomous decision-makers. The human-AI collaboration framework positioned LLMs as assistive technologies that augment rather than replace physician clinical judgment. In this framework, AI systems provided initial syndrome differentiation and treatment recommendations, which were then critically reviewed, modified, and approved by licensed TCM practitioners. Physicians retained full clinical authority and responsibility for final decisions.

This physician-supervised approach is necessary for several reasons. First, TCM clinical practice requires consideration of patient-specific factors and contextual elements that current AI systems cannot fully capture. Second, clinical responsibility and ethical accountability must remain with licensed health care providers who can integrate AI recommendations within the broader context of patient care. Third, the study results demonstrate that optimal outcomes were achieved through collaborative approaches where physicians leveraged AI capabilities while exercising critical oversight.

These findings should not be interpreted as suggesting readiness for autonomous AI deployment in TCM practice. Rather, they support the clinical value of AI as a decision support tool within physician-supervised frameworks, where human expertise guides and validates AI-generated recommendations.

### Limitations

This study has several important limitations that should be considered when interpreting the findings. First, the evaluation used examination cases from the TCM Practitioner Qualification Examination rather than real-time clinical encounters. While this methodological choice was necessary to ensure standardized evaluation and fair comparison across models, examination cases present with complete, well-organized information and predetermined diagnostic conclusions, whereas real clinical practice involves incomplete information, evolving presentations, and genuine diagnostic uncertainty. The structured format may favor certain AI reasoning patterns and lacks the dynamic elements of actual patient care, such as real-time communication, physical examination, and decision-making under time pressure. These factors suggest that the observed performance levels may not directly translate to real-world clinical settings, although this represents an essential first step before clinical validation studies.

Second, the clinical case evaluation sample size (n=30) was determined through stratified sampling to ensure representation across 7 major disease systems and balanced complexity distribution. Post hoc power analysis based on the observed large effect size (Cohen *f*=0.72, corresponding to mean score differences ranging from 10.02 to 17.31 on a 20-point scale) indicated that this sample size provided adequate statistical power (0.89) for detecting the substantial performance differences observed among the 5 models. The sample size was constrained by the substantial resources required for comprehensive expert evaluation (2 chief physician-level experts [Y. Wang and H. Shang] conducting detailed assessments across 7 dimensions per case). Future studies with larger, multi-institutional case sets would enhance confidence in the generalizability of these findings across the full spectrum of TCM pathologies, particularly for rare conditions and atypical presentations.

Third, despite implementing multiple quality control measures—including evaluator training, blinded evaluation, dual independent assessments, and structured scoring rubrics—the expert scoring methodology inherently involves subjective judgment. TCM clinical reasoning incorporates both explicit knowledge and tacit expertise that can be difficult to standardize completely across evaluators. The study demonstrated excellent interrater agreement between the 2 chief physician-level evaluators, suggesting these measures successfully limited variability. However, some degree of subjective judgment remains inherent to evaluating complex clinical reasoning, particularly in areas where multiple valid approaches may exist.

Additionally, the evaluation framework was based on contemporary TCM education standards and the TCM Practitioner Qualification Examination criteria, which prioritize evidence-based approaches and standardized diagnostic principles. While this framework ensures consistency and reproducibility in evaluation, it may not fully capture the theoretical diversity of different TCM schools (such as Shanghan school, Wenbing school, and various regional traditions) or individual practitioner variations in diagnostic and treatment preferences. The scoring rubrics emphasized core competencies that transcend school-specific approaches—including fundamental syndrome differentiation logic, treatment principle adherence, and medication safety—but may underrepresent the value of school-specific theoretical frameworks that experienced practitioners might preferentially use in clinical practice. Finally, this study was conducted in the context of China’s health care system, and findings may not directly generalize to TCM practice in other cultural contexts. The evaluation was also conducted at a specific point in time (March 2025) with models released in early 2025, representing a temporal snapshot of AI capabilities.

### Future Research Directions

Two critical research priorities emerge from these findings. First, large-scale prospective clinical trials in real-world TCM practice are essential to validate the human-AI collaboration benefits observed in this controlled evaluation, particularly regarding patient outcomes, diverse populations, and long-term effectiveness. Such trials should use rigorous randomized designs with clinically meaningful end points beyond decision quality metrics.

Second, multicenter collaborative research across different health care systems and cultural contexts is needed to assess generalizability and identify implementation considerations for broader clinical adoption, including practitioner training requirements, workflow integration, and patient acceptance across diverse traditional medicine settings.

### Conclusions

This study provides compelling evidence that LLMs, particularly DeepSeek-R1, demonstrate substantial capabilities in TCM knowledge assessment and clinical case analysis. The findings support the clinical value of human-AI collaboration, which achieved significant improvements in both decision-making quality (16.1% improvement) and efficiency (66.1% time reduction) compared with traditional approaches. The high system usability and favorable collaboration patterns indicate strong potential for successful integration into clinical practice.

These findings suggest that AI-assisted decision-making may address key challenges in TCM practice, including knowledge standardization, clinical training, and health care delivery efficiency. However, the study also highlights the continued importance of human clinical judgment, particularly in medication selection and individualized treatment formulation, where practitioner expertise remains essential.

For clinical practice, we recommend piloting human-AI collaboration systems in controlled clinical environments with appropriate training and support protocols. For policy development, these findings support investment in AI research and development specifically targeting traditional medicine applications, along with regulatory frameworks that ensure patient safety while facilitating innovation. The demonstrated benefits of this technology suggest that strategic implementation of AI assistance could significantly enhance the quality, efficiency, and accessibility of TCM care while preserving the fundamental principles and personalized approach that characterize this medical tradition.

## Supplementary material

10.2196/80167 Multimedia Appendix 1 160 basic knowledge test questions.

10.2196/80167 Multimedia Appendix 2 Detailed content of 30 case analyses.

10.2196/80167 Multimedia Appendix 3 Complete materials of 5 clinical cases.

10.2196/80167 Multimedia Appendix 4 Assessment scales and questionnaires.

10.2196/80167 Multimedia Appendix 5 Collaboration process records.

10.2196/80167 Multimedia Appendix 6 Detailed scoring results.

## Abbreviations

AI: artificial intelligence
ICC: intraclass correlation coefficient
LLM: large language model
OR: odds ratio
TCM: traditional Chinese medicine
