# Supplementary material for: Digitally Assisted Clinical Decision-Making in Traditional Chinese Medicine: Comparative Study of 5 Large Language Models
Source: JMIR Form Res. 2026 Mar 2;10:e80167. doi: 10.2196/80167 (PMC12954686; doi:10.2196/80167)
Supplement: Multimedia Appendix 1 [file formative-v10-e80167-s001.doc]

## Multimedia Appendix 1: 160 Basic Knowledge Test Questions

**Pulmonary System Diseases (15%) - 24 questions: Basic (37.5%) 9, Intermediate (37.5%) 9, Advanced (25%) 6**

1. 2023U3-8A1 Which of the following is NOT a characteristic of seasonal influenza?

A. High contagiousness B. Severe condition C. Similar symptoms D. Easy epidemic spread E. Little transformation

Answer: E

2. 2008U3-3A1 The main location of external pathogenic cough is:

A. Spleen B. Heart C. Lung D. Kidney E. Liver

Answer: C

3. 2019U3-119A1 Morning cough with paroxysmal aggravation, continuous heavy cough, with reduced cough after expectoration indicates:

A. Wind-heat cough B. Wind-cold cough C. Wind-dryness cough D. Phlegm-dampness cough E. Lung dryness and yin deficiency cough

Answer: D

4. 2023U3-10A1 For phlegm-heat cough, the preferred formula is:

A. Erchen Pingwei Powder B. Yuebi plus Banxia Decoction C. Sangbaipi Decoction D. Qingjin Huatan Decoction E. Sanzi Yangqin Decoction

Answer: D

5. 2019U3-118A1 The "dormant root" of asthma is:

A. Cold B. Heat C. Phlegm D. Qi E. Fire

Answer: C

6. 2019U3-2A1 For treating lung atrophy with deficiency-cold syndrome, the preferred formula is:

A. Mahuang Shengma Decoction B. Gancao Ganjiang Decoction C. Qingzao Jiufei Decoction D. Qiwei Duqi Pill E. Maimendong Decoction

Answer: B

7. 2021U3-21A1 The treatment principle for dyspnea with qi deficiency and yang collapse is:

A. Dispel phlegm and descend qi, diffuse lung and calm dyspnea B. Supplement lung, benefit qi and nourish yin C. Supplement lung and absorb kidney, descend qi and calm dyspnea D. Supplement kidney and absorb qi E. Support yang and secure collapse, sedate and absorb kidney qi

Answer: E

8. 2022U3-11A1 For patients with qi deficiency and dyspnea collapse, Shenfu Decoction is often taken with which medicine to secure collapse:

A. Bufei Pill B. Shenqi Pill C. Butian Dazao Pill D. Hexi Dan E. Yuehua Pill

Answer: D

9. 2015U3-50A1 For treating dyspnea with wind-cold blocking lung syndrome, the preferred formula is:

A. Shegan Mahuang Decoction B. Mahuang Decoction combined with Huagai Powder C. Erchen Decoction combined with Sanzi Yangqin Decoction D. Santao Decoction combined with Zhisou Powder E. Shengmai Powder combined with Bufei Decoction

Answer: B

10. 2020U3-27A1 For treating common cold with wind-heat attacking exterior syndrome, the preferred formula is:

A. Renshen Baidu Powder B. Congchi Jiegeng Decoction C. Jingfang Baidu Powder D. Xingjia Xiangru Drink E. Shensu Powder

Answer: B

11. 2008U3-5A1 For treating hot asthma during attack period, the preferred choice is:

A. Sangbaipi Decoction B. Maxing Shigan Decoction C. Suzi Jiangqi Decoction D. Dingchuan Decoction E. Xiebai Powder

Answer: D

12. 2024U3-19A1 Dry cough with short sound, or cough with small amount of sticky sputum, or sputum with blood streaks or spots, fresh red color, chest stuffiness with dull pain, afternoon heat in palms and soles, dry burning skin, dry mouth and throat, or slight night sweats; red tongue tip and border, thin coating, fine or rapid pulse. Syndrome type:

A. Lung yin deficiency B. Yin deficiency with fire hyperactivity C. Qi and yin deficiency D. Yin and yang deficiency E. Deficient fire scorching lung

Answer: A

13. 2020U3-34A1 The pathological basis of lung abscess formation and suppuration is:

A. Heat injuring lung qi B. Heat stagnation with blood stasis C. Flesh corruption and blood deterioration D. Heat toxin lingering E. Phlegm-stasis obstruction

Answer: B

14. 2023U3-23A1 Which of the following belongs to deficiency dyspnea symptoms:

A. Acute onset B. Coarse breathing with loud sound C. Deep inspiration for comfort D. Deep and prolonged breathing E. Accompanied by phlegm rales and cough

Answer: C

15. 2008U3-6A1 The key treatment principle for deficiency dyspnea is:

A. Supplement lung B. Strengthen spleen C. Absorb kidney D. Benefit qi E. Calm dyspnea

Answer: C

16. 2005U3-2A1 Among external pathogenic coughs, which one has protracted course and eventually leads to lung yin depletion:

A. Wind-cold B. Wind-heat C. Summer-dampness D. Wind-dryness E. Wind-dampness

Answer: D

17. 2001U3-2A1 For treating cough, the main focus should be on treating the lung, but attention should also be paid to treating:

A. Liver, spleen, kidney B. Heart, liver, kidney C. Heart, spleen, kidney D. Heart, liver, spleen E. Liver, stomach, kidney

Answer: A

18. 2019U3-116A1 Which of the following is INCORRECT as diagnostic basis for differentiating wind-cold and wind-heat:

A. Severity of aversion to cold and fever B. Thirst or no thirst C. Yellow or white tongue coating D. Floating or non-floating pulse E. Red, swollen, painful throat or not

Answer: D

19. 2020U3-11A1 The pathological key during asthma attack lies in:

A. Latent phlegm B. Lung failure in diffusion and descent C. Phlegm obstruction and qi closure D. Unclear lung qi E. Lung qi deficiency-cold

Answer: C

20. 2024U3-35A1 What are the characteristics of wind-phlegm asthma:

A. Wheezing sound like water fowl in throat, rapid breathing, dyspnea with qi rebellion, chest and diaphragm fullness like obstruction

B. Phlegm rales like roaring in throat, dyspnea with coarse qi surging, chest high with hypochondriac distension, cough attacks, sputum yellow or white, sticky and thick, difficult to expectorate

C. Wheezing sound in throat, chest and diaphragm vexation, rapid breathing, dyspnea with qi rebellion, unsatisfactory expectoration

D. Abundant phlegm saliva in throat, sound like sawing, dyspnea with chest fullness, can only sit but not lie down, sticky sputum difficult to expectorate

E. Wheezing like snoring in throat, low sound, shortness of breath, dyspnea worsened by movement, frequent attacks

Answer: D

21. 2006U3-28A1 Except for which item, all are causes of dyspnea:

A. External pathogen invasion B. Improper diet C. Emotional disturbance D. Abundant phlegm-heat E. Taxation and prolonged illness

Answer: D

22. 2011U3-59A1 The main pathological factor in early lung distension:

A. Blood stasis B. Water retention C. Phlegm turbidity D. Phlegm-stasis E. Qi stagnation

Answer: C

23. 2011U3-72A1 The best formula choice for treating lung consumption with qi and yin depletion:

A. Baozhen Decoction B. Baihe Gujin Pill C. Yuehua Pill D. Butian Dazao Pill E. Bufei Ejiao Decoction

Answer: A

24. 2019U3-22A1 Which of the following does NOT belong to the pathological manifestation stages of lung abscess:

A. Abscess formation stage B. Suppuration stage C. Exterior syndrome stage D. Ulceration stage E. Recovery stage

Answer: B

**Heart System Diseases (5%) - 8 questions: Basic (37.5%) 3, Intermediate (37.5%) 3, Advanced (25%) 2**

1. 2022U3-14A1 Among the following options, which belongs to the differential points between palpitation and severe palpitation:

A. Palpitation is persistent, severe palpitation is paroxysmal

B. Severe palpitation may transform into palpitation if not cured for long

C. Severe palpitation involves up-down surging, originating from lower abdomen

D. Palpitation cannot be controlled, worsened after activity

E. Palpitation mostly relates to emotions, severe palpitation can occur without mental factors

Answer: E

2. 2008U3-10A1 The main pathogenesis of chest impediment is:

A. Qi stagnation and blood stasis B. Cold congealing qi stagnation C. Phlegm-stasis interaction D. Yang qi deficiency E. Heart vessel impediment

Answer: E

3. 2000U3-3A1 The basic pathogenesis of insomnia is:

A. Root deficiency with branch excess, qi-blood imbalance B. Yang exuberance yin decline, yin-yang imbalance C. Yang qi insufficiency, internal yin-cold D. Qi-blood stagnation, heart vessel impediment E. Qi mechanism disorder, ascent-descent dysfunction

Answer: B

4. 2023U3-18A1 The fundamental pathogenesis of heart failure is:

A. Heart qi insufficiency B. Phlegm turbidity blocking C. Water retention attacking heart D. Stasis blocking heart vessels E. Heart deficiency with gallbladder timidity

Answer: A

5. 2005U3-70A1 For yin-cold congealing chest impediment with heart pain radiating to back, cold hands and feet, cold sweating, the preferred treatment is:

A. Tongmai Sini Decoction B. Huangqi Guizhi Wuwu Decoction C. Zhishi Xiebai Guizhi Decoction combined with Danggui Sini Decoction D. Jingui Shenqi Pill E. Gualou Xiebai Banxia Decoction

Answer: C

6. 2024U3-14A1 Regarding daily care of chest impediment, which of the following is INCORRECT:

A. Maintain smooth bowel movements, avoid tobacco and alcohol stimulation

B. Regulate emotions, avoid mental stimulation

C. Monitor tongue appearance, pulse, respiration, blood pressure, give oxygen when necessary

D. Maintain adequate sleep, persist in activities within capacity

E. Avoid cold, maintain air circulation

Answer: D

7. 2019U3-17A1 For yin deficiency fire hyperactivity type palpitation, if accompanied by five-center heat, nocturnal emission and lumbar soreness, additional treatment should include:

A. Tianwang Buxin Dan B. Liuwei Dihuang Pill C. Maiwei Dihuang Pill D. Zhibai Dihuang Pill E. Jinsuo Gujing Pill

Answer: D

8. 2020U3-30A1 Which of the following does NOT belong to the causes of heart failure:

A. Prolonged illness consuming injury B. External pathogen invasion C. Seven emotions injury D. Taxation internal injury E. Blood stasis impediment

Answer: E

**Brain System Diseases (10%) - 16 questions: Basic (37.5%) 6, Intermediate (37.5%) 6, Advanced (25%) 4**

1. 2021U3-32A1 Headache involving forehead and eyebrow ridge belongs to:

A. Taiyang headache B. Jueyin headache C. Yangming headache D. Shaoyang headache E. Taiyin headache

Answer: C

2. 2024U3-37A1 The main formula for treating vertigo with phlegm turbidity veiling clear:

A. Tianma Gouteng Drink B. Banxia Baizhu Tianma Decoction C. Guipi Decoction D. Zuogui Pill E. Erchen Pingwei Powder

Answer: B

3. 2014U3-46A1 Symptoms of persistent headache with fixed pain location, stabbing pain, or history of head trauma, purple tongue, fine pulse. The best formula for treatment is:

A. Taohong Siwu Decoction B. Tongqiao Huoxue Decoction C. Xuefu Zhuyu Decoction D. Fuyuan Huoxue Decoction E. Shixiao Powder

Answer: B

4. 2011U3-45A1 For treating vertigo with phlegm turbidity blocking middle, the preferred choice is:

A. Tianma Gouteng Drink B. Banxia Baizhu Tianma Decoction C. Zhengan Xifeng Decoction D. Buyang Huanwu Decoction E. Dihuang Drink

Answer: B

5. 2020U3-18A1 Stroke affecting zang-fu requires differentiation of:

A. External vs. internal injury B. Deficiency vs. excess nature C. Closed vs. collapsed syndrome D. Meridian vs. zang-fu involvement E. Severity of condition

Answer: C

6. 2006U3-24A1 Except for which item, all are characteristics of stroke closed syndrome:

A. Sudden collapse B. Tightly clenched teeth C. Mouth cannot open D. Rigid limbs E. Incontinence of urine and feces

Answer: E

7. 2006U3-18A1 For yangming headache, the guiding meridian drugs that can be selected are:

A. Qianghuo, Manjingzi B. Qianghuo, Chuanxiong C. Gegen, Baizhi D. Chaihu, Chuanxiong E. Wuzhuyu, Gaoben

Answer: C

8. 2020U3-7A1 Which of the following does NOT belong to the causes of vertigo:

A. Emotional disturbance B. Dietary irregularity C. Trauma from falls D. Advanced age with kidney deficiency E. External wind-dampness invasion

Answer: E

9. 2022U3-15A1 Common pathological factors of vertigo include:

A. Wind, phlegm B. Wind, fire, phlegm C. Qi, fire, phlegm, stasis D. Wind, fire, phlegm, qi E. Wind, fire, phlegm, stasis

Answer: E

10. 2023U3-9A1 The main differential point between stroke and syncope is:

A. Presence of sequelae B. Presence of tightly clenched teeth C. Presence of drooling foam D. Presence of limb reversal E. Presence of spirit clouding

Answer: A

11. 2005U3-36A1 For treating stroke with qi deficiency blood stagnation and meridian vessel stasis obstruction, the preferred choice is:

A. Tianma Gouteng Decoction B. Buyang Huanwu Decoction C. Taohong Siwu Decoction D. Zhengan Xifeng Decoction E. Dahualuo Dan with Erchen Decoction

Answer: B

12. 2023U3-29A1 The formula used for treating mania with fire exuberance and yin injury syndrome:

A. Shengtieluo Drink B. Diankuang Mengxing Decoction C. Eryin Decoction combined with Hupo Yangxin Dan D. Shunqi Daotan Pill E. Yangxin Decoction combined with Yueju Pill

Answer: C

13. 2005U3-5A1 The main disease location of internal injury headache is:

A. Liver, spleen, stomach B. Liver, heart, spleen C. Liver, spleen, kidney D. Heart, liver, kidney E. Gallbladder, liver, spleen

Answer: C

14. 2021U3-12A1 In syndrome differentiation and treatment of vertigo, the first differentiation should be:

A. External vs. internal injury B. Cold, heat, deficiency, excess C. Deficiency, excess, slow, urgent D. Root and branch deficiency-excess E. Disease-affected organs

Answer: E

15. 2006U3-39A1 The pathological basis of stroke is:

A. Wind, fire, phlegm, stasis B. Qi-blood counterflow disorder C. Heart-liver fire hyperactivity D. Liver yang hyperactivity E. Liver-kidney yin deficiency

Answer: E

16. 2006U3-44A1 For treating mania with phlegm-heat stasis binding syndrome, the preferred choice is:

A. Shunqi Daotan Decoction B. Yueju Pill C. Shengtieluo Drink D. Hupo Yangxin Dan E. Diankuang Mengxing Decoction

Answer: E

**Spleen-Stomach System Diseases (15%) - 24 questions: Basic (37.5%) 9, Intermediate (37.5%) 9, Advanced (25%) 6**

1. 2005U3-4A1 The organs most closely related to stomach pain are:

A. Liver, spleen, gallbladder B. Liver, spleen, stomach C. Liver, spleen, kidney D. Lung, spleen, stomach E. Lung, liver, stomach

Answer: B

2. 2006U3-10A1 For treating stomach pain with spleen-stomach deficiency-cold syndrome, the preferred choice is:

A. Xiaojianzhong Decoction B. Lizhong Pill C. Fuzi Lizhong Pill D. Liangfu Pill E. Huangqi Jianzhong Decoction

Answer: E

3. 2001U3-14A1 The disease location of vomiting is:

A. Intestine, liver, spleen B. Stomach, liver, spleen C. Spleen, stomach, lung D. Lung, stomach, kidney E. Liver, stomach, intestine

Answer: B

4. 2006U3-37A1 The key organ in hiccup pathological changes is:

A. Liver B. Spleen C. Lung D. Stomach E. Gallbladder

Answer: D

5. 2023U3-17A1 Which of the following does NOT belong to pathological factors of abdominal pain:

A. Cold congealing B. Blood stasis C. Fire constraint D. Wind pathogen E. Food accumulation

Answer: D

6. 2008U3-27A1 For treating cold-dampness dysentery, the preferred choice is:

A. Buhuanjin Zhengqi Powder B. Taohua Decoction C. Lianli Decoction D. Huangshi Decoction E. Zhenren Yangzang Decoction

Answer: A

7. 2015U3-71A1 The treatment principle for qi deficiency constipation is:

A. Clear heat and moisten dryness B. Smooth qi and guide stagnation C. Benefit qi and moisten intestines D. Nourish blood and moisten intestines E. Warm yang and unblock

Answer: C

8. 2015U3-60A1 The clinical characteristics of cold constipation are:

A. Stool not dry, lumbar and knee soreness B. Dry hard stool, desire to defecate but cannot C. Stool not dry, straining without strength D. Difficult astringent stool, cold hands and feet E. Dry hard stool, dry mouth and bad breath

Answer: D

9. 2011U3-54A1 Elderly female with difficult astringent stool, powerless defecation, lumbar and knee soreness, aversion to cold with cold limbs, pale tongue, white coating, deep fine pulse, should select:

A. Runchang Pill B. Jichuan Decoction C. Huangqi Decoction D. Buzhong Yiqi Decoction E. Wuren Pill

Answer: B

10. 2019U3-8A1 Which of the following is meaningless for differentiating stomach pain from true heart pain:

A. Presence of radiating pain B. Nature of pain C. Presence of aversion to cold and fever D. Presence of belching, acid regurgitation, noisy stomach E. Presence of palpitation, shortness of breath, sweating, cold limbs

Answer: C

11. 2022U3-26A1 For stomach pain with phlegm-dampness blocking middle syndrome, the formula is Erchen Decoction plus:

A. Cangzhu, Houpo B. Huoxiang, Dafupi C. Baizhu, Shengjiang D. Mahuang, Kuandonghua E. Sangbaipi, Tinglizi

Answer: A

12. 2022U3-5A1 For treating stomach focal distension with dampness-heat blocking stomach syndrome, the preferred formula is:

A. Sanren Decoction B. Lianpo Drink C. Zuojin Pill D. Longdan Xiegan Decoction E. Qingwei Powder

Answer: B

13. 2024U3-33A1 In syndrome differentiation of vomiting, the first differentiation should be:

A. Deficiency-excess B. Cold-heat C. Root-branch D. Exterior-interior E. Disease location

Answer: A

14. 2008U3-21A1 For treating hiccup with qi mechanism constraint syndrome, the preferred choice is:

A. Dingxiang Powder B. Yiwei Decoction C. Wumo Drink D. Zhuye Shigao Decoction E. Jupi Zhuru Decoction

Answer: C

15. 2019U3-117A1 For treating abdominal pain with dampness-heat stagnation syndrome, the preferred formula is:

A. Dachengqi Decoction B. Zhengqi Tianxiang Powder C. Zhishi Daozhiwan D. Xiaojianzhong Decoction E. Dahuang Fuzi Decoction

Answer: A

16. 2000U3-16A1 The important factor in diarrhea onset is:

A. Liver-spleen disharmony B. Spleen-stomach weakness C. Spleen deficiency with dampness exuberance D. Spleen-kidney yang deficiency E. Spleen failure in transportation

Answer: C

17. 2015U3-85A1 For treating dysentery cold-dampness type, the preferred formula is:

A. Shenling Baizhu Powder B. Huoxiang Zhengqi Powder C. Buhuanjin Zhengqi Powder D. Baohe Pill E. Zhengqi Tianxiang Powder

Answer: C

18. 2015U3-81A1 Which does NOT belong to characteristics of spleen deficiency diarrhea stool:

A. Loose watery stool B. Light colored stool C. Not very foul odor D. Diarrhea after eating E. Much mucus in stool

Answer: E

19. 2005U1-14A1 Which of the following is NOT a manifestation of stomach pain with spleen-stomach deficiency-cold syndrome:

A. Dull stomach pain, likes warmth and pressure B. Empty stomach pain worse, pain reduced with food C. Cold hands and feet D. Pain increased with cold, sudden onset E. Vomiting clear water

Answer: D

20. 2021U3-3A1 The common symptom of dysphagia-occlusion and vomiting is:

A. Abdominal pain B. Belching C. Vomiting D. Difficult swallowing E. No desire for food

Answer: C

21. 2021U3-4A1 For treating dampness-heat dysentery with resolved exterior syndrome but persistent dysentery, the formula to select is:

A. Huoren Baidu Powder B. Gegen Qinlian Decoction C. Xianglian Pill D. Baitouweng Decoction E. Shaoyao Decoction

Answer: C

22. 2011U3-71A1 For chronic dysentery with extremely deficient spleen yang, cold accumulation in intestines not transforming, triggered by cold, white jelly-like discharge, fatigue with poor appetite, pale tongue with white coating, deep pulse, can use:

A. Wenpi Decoction B. Weiling Decoction C. Lianli Decoction D. Taohua Decoction E. Buzhong Yiqi Decoction

Answer: A

23. 2019U3-13A1 For heat constipation with mild dryness-heat or unsatisfactory bowel movement after medication, can select:

A. Gengyi Pill B. Qinglin Pill C. Dachengqi Decoction D. Maziren Pill E. Runchang Pill

Answer: B

24. 2008U3-28A1 For blood deficiency constipation syndrome with recovered yin-blood but still dry stool, the preferred treatment is:

A. Huangqi Decoction B. Zengye Decoction C. Runchang Pill D. Wuren Pill E. Qinglin Pill

Answer: D

**Liver-Gallbladder System Diseases (15%) - 24 questions: Basic (37.5%) 9, Intermediate (37.5%) 9, Advanced (25%) 6**

1. 2008U3-29A1 The key pathological factor in jaundice formation is:

A. Heat pathogen B. Cold pathogen C. Epidemic toxin D. Blood stasis E. Dampness pathogen

Answer: E

2. 2008U3-30A1 For treating jaundice yin-jaundice syndrome, the preferred choice is:

A. Mahuang Lianqiao Chixiaodou Decoction B. Zhizi Baipi Decoction C. Yinchen Wuling Powder D. Yinchen Shu Fu Decoction E. Yinchenhao Decoction

Answer: D

3. 2024U3-25A1 Which is NOT a characteristic of accumulation syndrome:

A. Lumps gather and disperse irregularly B. Disease in qi division C. Pain without fixed location D. Mostly zang disease E. Mainly distending pain

Answer: D

4. 2021U3-19A1 Which does NOT belong to characteristics of amassment syndrome:

A. Palpable when touched B. Fixed and immobile C. Pain with fixed location D. Mainly stabbing pain E. Disease in qi division

Answer: E

5. 2020U3-35A1 The best treatment formula for liver qi stagnation type accumulation syndrome is:

A. Wuji Powder B. Jinlingzi Powder C. Shixiao Powder D. Xiaoyao Powder, Muxiang Shunqi Powder E. Chaihu Shugan Powder

Answer: D

6. 2022U3-4A1 Yiguan Decoction mainly treats:

A. Goiter with heart-liver yin deficiency syndrome B. Goiter with liver fire hyperactivity syndrome C. Goiter with qi constraint phlegm obstruction syndrome D. Drum distension with qi stagnation dampness obstruction syndrome E. Drum distension with water-heat accumulation syndrome

Answer: A

7. 2022U3-37A1 The basic pathogenesis of hypochondriac pain is:

A. Liver network disharmony B. Liver qi stagnation C. Blood stasis blocking network D. Dampness-heat accumulation E. Liver fire ascending

Answer: A

8. 2006U3-45A1 For treating jaundice with heat predominant over dampness syndrome, the preferred choice is:

A. Yinchenhao Decoction B. Yinchen Wuling Powder C. Dachaihu Decoction D. Xijiao Powder E. Yinchen Shufu Decoction

Answer: A

9. 2015U3-113B1 For treating hypochondriac pain with liver-gallbladder dampness-heat syndrome, the preferred formula is:

A. Longdan Xiegan Decoction B. Chaihu Shugan Powder C. Xuefu Zhuyu Decoction D. Yiguan Decoction E. Yinchenhao Decoction

Answer: A

10. 2011U3-52A1 The basic pathogenesis of hypochondriac pain is:

A. Dampness-heat internal accumulation B. Qi stagnation blood stasis C. Liver qi stagnation D. Liver network disharmony E. None of the above

Answer: D

11. 2015U3-56A1 Which of the following does NOT belong to jaundice syndrome differentiation points:

A. Differentiate yang-jaundice, yin-jaundice B. Differentiate jaundice severity C. Differentiate jaundice location D. Differentiate yin-jaundice causes E. Differentiate yang-jaundice dampness-heat severity

Answer: C

12. 2024U3-22A1 For treating jaundice yang-jaundice with heat predominant over dampness, the preferred formula is:

A. Tiaoying Drink combined with Xuefu Zhuyu Decoction B. Yinchen Wuling Powder combined with Ganlu Xiaodu Dan C. Chaihu Shugan Powder combined with Weiling Decoction D. Zhongman Fenxiao Pill combined with Yinchenhao Decoction E. Yinchenhao Decoction

Answer: E

13. 2015U3-59A1 The main treatment formula for jaundice gallbladder constraint heat is:

A. Xiaochaihu Decoction B. Dachaihu Decoction C. Gegen Qinlian Decoction D. Shengma Gegen Decoction E. Longdan Xiegan Decoction

Answer: B

14. 2006U3-17A1 For treating drum distension with water-dampness trapped spleen syndrome, the preferred choice is:

A. Chaihu Shugan Powder combined with Weiling Decoction B. Shipi Drink C. Zhongman Fenxiao Pill D. Tiaoying Drink E. Fuzi Liling Decoction

Answer: B

15. 2015U3-52A1 After jaundice subsides, if the body still has qi stagnation blood stasis, should select:

A. Dahuang Zhechong Pill B. Xiaoyao Powder combined with Biejia Pill C. Xuefu Zhuyu Decoction D. Shentong Zhuyu Decoction E. Fuyuan Huoxue Decoction

Answer: B

16. 2022U3-28A1 Which of the following statements about amassment syndrome is INCORRECT:

A. Disease in blood division B. Pain fixed and immobile, pain with fixed location C. Mostly zang disease D. Pathogenesis is qi mechanism constraint E. Mainly stabbing pain

Answer: D

17. 2011U3-58A1 The treatment principle for middle stage of amassment and accumulation:

A. Attack pathogen, move qi and activate blood B. Support healthy and cultivate root C. Attack and supplement simultaneously D. Regulate qi E. Soothe liver and regulate qi

Answer: C

18. 2021U3-28A1 The main disease location of drum distension is:

A. Liver, spleen, kidney B. Liver, gallbladder, spleen C. Liver, lung, spleen, kidney D. Liver, gallbladder, spleen, kidney E. Liver, gallbladder, spleen, stomach

Answer: A

19. 2020U3-5A1 Which does NOT belong to pathological factors of jaundice:

A. Phlegm turbidity B. Blood stasis C. Warm pathogen D. Epidemic toxin E. Qi stagnation

Answer: A

20. 2008U3-100A1 Jaundice patient with yellow body and eyes, heavy head and tired body, chest and epigastric focal distension, decreased appetite, nausea and vomiting, loose stool, thick greasy slightly yellow tongue coating, soggy rapid pulse. Treatment should preferably select:

A. Dachaihu Decoction B. Xiaochaihu Decoction C. Yinchen Wuling Powder combined with Ganlu Xiaodu Dan D. Yinchenhao Decoction E. Xijiao Powder

Answer: C

21. 2006U3-16A1 Which of the following does NOT belong to causes of amassment and accumulation:

A. Emotional disturbance B. Dietary damage C. Cold pathogen invasion D. Post-illness condition E. Trauma injury

Answer: E

22. 2017U2-45A1 Which of the following does NOT belong to clinical manifestations of amassment syndrome:

A. Poor spirits B. Thin body shape C. No desire for food D. Alternating constipation and diarrhea E. Old person appearance

Answer: D

23. 2009U3-92A1 Accumulation syndrome patient with food stagnation phlegm obstruction, heavy phlegm-dampness, after taking Wumo Decoction, bowel qi is smooth but symptoms not reduced, white greasy tongue coating not transforming. Treatment should preferably select:

A. Erchen Decoction B. Huopu Xialing Decoction C. Pingwei Powder D. Wuling Powder E. Xiangsu Powder

Answer: C

24. 2011U3-48A1 Which of the following does NOT belong to pathological factors of drum distension:

A. Cold congealing B. Qi stagnation C. Water-dampness D. Blood stasis E. Water fluid

Answer: A

**Kidney System Diseases (15%) - 24 questions: Basic (37.5%) 9, Intermediate (37.5%) 9, Advanced (25%) 6**

1. 2001U3-21A1 The organs involved in edema pathogenesis are:

A. Heart, liver, spleen B. Liver, spleen, kidney C. Lung, spleen, kidney D. Spleen, kidney, heart E. Kidney, heart, lung

Answer: C

2. 2021U3-31A1 The treatment principle for yin edema is:

A. Expel pathogen and drive out water, clear heat and detoxify B. Promote sweating and diuresis, regulate qi and transform dampness C. Support healthy as main, strengthen spleen and warm kidney D. Expel pathogen and diuresis, transport spleen and transform dampness E. None of the above

Answer: C

3. 2008U3-33A1 The treatment principle for yang edema wind-water mutual struggle syndrome is:

A. Disperse wind and clear heat, diffuse lung and move water B. Diffuse lung and detoxify, diuresis and reduce swelling C. Strengthen spleen and transform dampness, warm yang and diuresis D. Warm and transport spleen yang to facilitate water-dampness E. Separate and diuresis dampness-heat, attack and purge to drive water

Answer: A

4. 2022U3-13A1 The main pathological factor belonging to strangury syndrome is:

A. Phlegm turbidity B. Blood stasis C. Qi stagnation D. Dampness-heat E. Healthy deficiency

Answer: D

5. 2001U3-7A1 The differential diagnosis between hematuria and blood strangury mainly lies in:

A. Depth of urine color B. Amount of urine C. Urine odor condition D. Presence of urinary pain E. None of the above

Answer: D

6. 2008U3-36A1 For treating stone strangury, the preferred choice is:

A. Chengshi Bixie Fenqing Drink B. Wubi Shanyao Pill C. Bazheng Powder D. Chenxiang Powder E. Shiwei Powder

Answer: E

7. 2005U1-45A1 For treating strangury syndrome with yin deficiency fire hyperactivity, the preferred choice is:

A. Liuwei Dihuang Decoction B. Dabu Pill C. Zhibai Dihuang Decoction D. Zuogui Pill E. Qinghao Biejia Decoction

Answer: C

8. 2022U3-35A1 The common characteristic of urinary retention and uroschesis is:

A. Urinary blockage B. Vomiting C. Lower abdominal distension and fullness D. Limb twitching E. Skin itching

Answer: A

9. 2024U3-32A1 The formula for treating urinary retention lung heat abundant syndrome is:

A. Bazheng Powder B. Chenxiang Powder C. Didang Pill D. Qingfei Drink E. Chunze Decoction

Answer: D

10. 2013U3-19A1 The key disease location of edema is:

A. Heart B. Liver C. Lung D. Spleen E. Kidney

Answer: E

11. 2023U3-25A1 Edema from bottom to top, then whole body, swollen areas with loose skin, depressed when pressed and difficult to recover, even like mud, syndrome belongs to:

A. Wind pathogen B. Damp toxin C. Dampness-heat D. Kidney deficiency E. Phlegm-dampness

Answer: D

12. 2023U3-22A1 Which belongs to the treatment principle of yin edema:

A. Expel pathogen as main B. Support healthy as main C. Attack first then supplement D. Promote sweating and diuresis E. Purge and drive water

Answer: B

13. 2015U3-22A1 Which of the following does NOT belong to clinical manifestations of edema wind-water mutual struggle syndrome:

A. Onset often preceded by cold and sore throat B. Edema starts from eyelids and face C. Edema spreads rapidly throughout body D. Reduced urine volume often with hematuria E. Heavy edema with difficult recovery when pressed

Answer: E

14. 2021U3-13A1 The differential point between strangury and urinary retention is:

A. Presence of frequent urination B. Presence of difficult urination C. Presence of turbid urine D. Presence of urethral pain E. Presence of hematuria

Answer: D

15. 2008U3-34A1 The pathogenesis most closely related to stone strangury pathogenesis is:

A. Spleen deficiency with middle qi sinking B. Kidney deficiency with lower origin not secure C. Dampness-heat accumulating in lower burner D. Heat exuberance forcing blood to move recklessly E. Qi constraint transforming fire and injuring yin

Answer: C

16. 2019U3-30A1 For blood strangury kidney yin deficiency syndrome treatment should select:

A. Guipi Decoction plus thistle, eclipta B. Xiaoji Drink C. Zhibai Dihuang Pill plus thistle, donkey-hide gelatin D. Xiaoji Drink combined with Daozhisan E. Wubi Shanyao Pill

Answer: C

17. 2008U3-37A1 Although urinary retention disease location is mainly in bladder and kidney, the organs closely related to this disease also include:

A. Lung, spleen, stomach, triple burner B. Lung, kidney, stomach, triple burner C. Liver, spleen, stomach, small intestine D. Lung, spleen, liver, triple burner E. Lung, spleen, liver, small intestine

Answer: D

18. 2022U3-18A1 The preferred formula for impotence with kidney gate fire decline syndrome is:

A. Zanyu Pill B. Zuogui Pill C. Yougui Pill D. Qiyang Yuxin Dan E. Jisheng Shenqi Pill

Answer: A

19. 2005U3-12A1 Which of the following is NOT a common cause of edema onset:

A. Wind pathogen external attack B. Damp toxin invasion C. Dietary taxation D. Sexual excess E. Emotional internal injury

Answer: E

20. 2011U3-12A1 Edema syndrome caused by spleen yang not transporting, transportation powerless, belongs to:

A. Deficiency with excess inside B. Excess with deficiency inside C. Deficiency transforming to excess D. Excess transforming to deficiency E. Extreme deficiency appearing excess

Answer: A

21. 2017U2-4A1 Which of the following diseases belongs to edema complications:

A. Focal distension B. Diabetes C. Jaundice D. Numbness E. Chest impediment

Answer: E

22. 2008U3-35A1 Except for which item, all are common manifestations of various strangunary syndromes:

A. Frequent urgent urination B. Lumbar soreness C. Dripping astringent pain D. Hematuria with pain E. Lower abdominal urgency

Answer: D

23. 2023U3-11A1 Which belongs to common points of strangury and urinary retention:

A. Frequent urination B. Astringent painful urination C. Hematuria D. Reduced urine volume E. Water retention in bladder

Answer: D

24. 2024U3-16A1 The disease location of impotence is:

A. Ancestral sinew B. Kidney C. Heart D. Spleen E. Liver

Answer: A

**Qi-Blood-Body Fluid Diseases (15%) - 24 questions: Basic (37.5%) 9, Intermediate (37.5%) 9, Advanced (25%) 6**

1. 2024U3-24A1 Depression syndrome pathogenesis is most closely related to which organ:

A. Liver B. Heart C. Spleen D. Lung E. Kidney

Answer: A

2. 2001U3-74A1 Depression syndrome patient with throat discomfort, like something obstructing, cannot cough out or swallow down, chest stuffiness, white greasy tongue coating, wiry slippery pulse. The syndrome is:

A. Phlegm-qi constraint B. Liver qi stagnation C. Qi constraint transforming fire D. Phlegm turbidity disturbing above E. Worry injuring spirit

Answer: A

3. 2014U3-41A1 Which of the following is the main syndrome of stomach heat blazing type nosebleed:

A. Headache and vertigo B. Red eyes C. Irritability and anger D. Wiry rapid pulse E. Dry mouth with foul breath

Answer: E

4. 2023U3-5A1 Fluid retention in limbs is called:

A. Phlegm retention B. Water retention C. Suspended fluid D. Branch fluid E. Overflow fluid

Answer: E

5. 2015U3-30A1 The main formula for treating diabetes lung heat fluid injury syndrome is:

A. Xiaoke Formula B. Yunü Decoction C. Liuwei Dihuang Pill D. Jingui Shenqi Pill E. Zhenwu Decoction

Answer: A

6. 2022U3-3A1 Qinggu Powder mainly treats which internal injury fever syndrome type:

A. Yin deficiency fever B. Blood deficiency fever C. Qi deficiency fever D. Yang deficiency fever E. Phlegm-dampness fever

Answer: A

7. 2003U3-102A1 Patient with low fever, dizziness; palpitation unease, pale complexion, pale lips and nails, pale tongue, fine pulse. The treatment principle is:

A. Benefit qi and nourish blood B. Benefit qi and strengthen spleen C. Nourish yin and clear heat D. Activate blood and transform stasis E. Soothe liver and clear heat

Answer: A

8. 2002U3-102A1 Consumptive disease patient with shortness of breath and spontaneous sweating, low weak voice, alternating chills and fever, usually susceptible to colds, pale tongue, weak pulse. The syndrome is:

A. Lung qi deficiency B. Spleen qi deficiency C. Lung yin deficiency D. Spleen yang deficiency E. Kidney qi deficiency

Answer: A

9. 2001U3-65A1 Consumptive disease patient with dry mouth and lips, no desire for food, dry constipation, even dry retching and hiccup, flushed complexion, red dry tongue with scanty coating, fine rapid pulse. The syndrome is:

A. Lung yin deficiency B. Spleen-stomach yin deficiency C. Liver yin deficiency D. Kidney yin deficiency E. Heart yin deficiency

Answer: B

10. 2000U3-10A1 The disease location of depression syndrome is mostly in:

A. Heart, spleen, stomach B. Liver, spleen, stomach C. Liver, spleen, kidney D. Liver, stomach, kidney E. Heart, liver, spleen

Answer: E

11. 2000U3-6A1 External pathogen invasion damaging vessels causing bleeding, the most common pathogen is:

A. Wind B. Cold C. Summer-heat D. Heat E. Dryness

Answer: D

12. 2011U3-22A1 The best formula choice for treating nosebleed heat pathogen attacking lung syndrome is:

A. Yinqiao Powder B. Shashen Maidong Decoction C. Xiebai Powder D. Sangju Drink E. Qingzao Jiufei Decoction

Answer: D

13. 2011U3-19A1 Nosebleed with fresh red blood, thirst and desire to drink, dry nose, dry mouth with foul breath, irritability, constipation, red tongue, yellow coating, rapid pulse, the syndrome differentiation is:

A. Liver fire attacking lung syndrome B. Yin deficiency fire hyperactivity syndrome C. Vexation injuring lung syndrome D. Liver fire ascending syndrome E. Stomach heat blazing syndrome

Answer: E

14. 2008U3-41A1 The treatment principle of phlegm retention is:

A. Diffuse lung B. Strengthen spleen C. Warm transformation D. Supplement kidney E. Promote sweating

Answer: C

15. 2014U3-40A1 The main diseased organs of diabetes are:

A. Lung, spleen, kidney B. Lung, stomach, kidney C. Heart, liver, kidney D. Liver, spleen, kidney E. Spleen, stomach, kidney

Answer: B

16. 2020U3-31A1 Which of the following descriptions about sweating syndrome pathogenesis is INCORRECT:

A. Spontaneous sweating mostly due to qi deficiency B. Night sweating mostly due to yin deficiency C. Pathological nature mostly deficiency with little excess D. Excess patterns mostly due to external wind-heat and liver fire E. Basic pathogenesis is yin-yang imbalance, interstices not secure, nutrient-defensive disharmony

Answer: D

17. 2001U3-27A1 Treating consumptive disease should mainly supplement which of the following:

A. Heart, kidney B. Heart, lung C. Lung, kidney D. Spleen, kidney E. Liver, kidney

Answer: D

18. 2002U3-26A1 For treating consumptive disease spleen-stomach yin deficiency, the preferred choice is:

A. Yunü Decoction B. Yiwei Decoction C. Shashen Maidong Decoction D. Maimendong Decoction E. Yiguan Decoction

Answer: B

19. 2005U3-13A1 The general treatment principle of depression syndrome is:

A. Soothe liver and resolve depression B. Transform phlegm and scatter binding C. Nourish heart and calm spirit D. Nourish blood and soften liver E. Unblock qi mechanism

Answer: E

20. 2006U3-22A1 For treating hematemesis stomach heat abundant syndrome, the preferred choice is:

A. Yunü Decoction B. Longdan Xiegan Decoction C. Modified Qingwei Powder combined with Xiexin Decoction D. Diyu Powder combined with Huaijiao Pill E. Xiexin Decoction combined with Shihui Powder

Answer: E

21. 2019U3-3A1 For treating branch fluid with much fluid little cold, no exterior syndrome, dyspnea cough with thin sputum or cannot rest, chest fullness with qi rebellion, should select:

A. Xiaoqinglong Decoction B. Lingguizhugan Decoction C. Linggan Wuwei Jiangxin Decoction D. Tingli Dazao Xiefei Decoction E. Zhenwu Decoction

Answer: D

22. 2005U3-17A1 For diabetes lower burner, if yin-yang deficiency appears, treatment should preferably select:

A. Buzhong Yiqi Decoction B. Liuwei Dihuang Pill C. Guipi Decoction D. Jingui Shenqi Pill E. Qiwei Baizhu Powder

Answer: D

23. 2024U3-23A1 For treating lung defensive not secure sweating syndrome with half-body sweating, add which drugs:

A. Maidong, Wuweizi B. Fuxiaomai, Gancao C. Dangshen, Baizhu D. Guizhi, Baishao E. Fuzi, Shengjiang

Answer: B

24. 2023U3-13A1 Which does NOT belong to main causes of internal injury fever:

A. Qi deficiency B. Blood heat C. External trauma D. Blood stasis E. Emotional disturbance

Answer: B

**Limb and Meridian Diseases (10%) - 16 questions: Basic (37.5%) 6, Intermediate (37.5%) 6, Advanced (25%) 4**

1. 2000U3-21A1 The clinical characteristics of painful impediment are:

A. Joint soreness and pain, wandering and unfixed B. Joint pain with red swelling and burning heat C. Severe pain with fixed location D. Heavy soreness and pain, skin numbness E. Inflexible extension-flexion, palpitation and shortness of breath

Answer: C

2. 2002U3-22A1 For treating wandering impediment, the preferred choice is:

A. Wutou Decoction B. Yiyiren Decoction C. Fangfeng Decoction D. Xuanbi Decoction E. Baihu plus Guizhi Decoction

Answer: C

3. 2022U3-19A1 The disease location of atrophy syndrome is in:

A. Sinews and vessels, muscles B. Bones C. Spleen-stomach D. Ancestral sinews E. Heart, liver, kidney

Answer: A

4. 2006U3-40A1 For treating atrophy syndrome liver-kidney deficiency, the preferred choice is:

A. Huqian Pill B. Shengyu Decoction C. Lujiaojiao Pill D. Buxue Rongjin Pill E. Duhuo Jisheng Decoction

Answer: A

5. 2011U4-40A1 The main formula for cold-dampness lumbar pain is:

A. Simiao Pill B. Zuogui Pill C. Yougui Pill D. Ganjiang Ling Zhu Decoction E. Qing'e Pill

Answer: D

6. 2013U3-37A1 For treating dampness-heat lumbar pain, the preferred choice is:

A. Ganjiang Lingzhu Decoction B. Simiao Pill C. Qianghuo Shengshi Decoction D. Yiyiren Decoction E. Wutou Decoction

Answer: B

7. 2006U3-31A1 For treating chronic impediment where wind, cold, dampness predominance not obvious, the formula that can be selected is:

A. Fangfeng Decoction B. Yiyiren Decoction C. Xuanbi Decoction D. Juanbi Decoction E. Wutou Decoction

Answer: D

8. 2000U3-7A1 Chronic impediment causing palpitation, the main pathogenesis is:

A. Heart yang not vibrant B. Blood stasis blocking collaterals C. Water retention attacking heart D. Heart blood insufficiency E. Yin deficiency fire hyperactivity

Answer: B

9. 2020U3-16A1 The basic pathogenesis of lumbar pain disease is:

A. Wind, cold, dampness pathogens blocking meridians B. Sinew vessel impediment, lumbar mansion losing nourishment C. Qi stagnation blood stasis, meridians blocked D. Taxation injury sprain, meridians blocked E. Kidney essence deficiency, lumbar mansion losing nourishment

Answer: B

10. 2024U3-139B1 For impediment syndrome lower limb pain, the drugs that should be combined are:

A. Duhuo, Chuanniuxi, Mugua B. Tubeimu, Maoyanggcao, Fengfang, Weilingxian C. Tufuling, Cheqianzi, Yiyiren, Maozhuacao D. Jianghuang, Guizhi, Qianghuo E. Gegen, Shenjincao, Guizhi, Qianghuo

Answer: A

11. 2022U3-97A2 Patient, male, 59 years old, fever subsided but limb weakness and powerlessness, muscle wasting, vexation and thirst, dry throat with discomfort, yellow scanty urine, dry stool. Red tongue, yellow coating, fine rapid pulse. Should select:

A. Modified Ermiao Powder B. Qingzao Jiufei Decoction C. Weiling Decoction D. Yiwei Decoction E. Sangxing Decoction

Answer: B

12. 2019U3-57A2 Zhao, female, 52 years old. Started feeling lower limb weakness half year ago, gradually unable to stand, lumbar spine fatigue, dizziness and tinnitus, dry mouth and tongue, red tongue with scanty coating, deep fine rapid pulse. Which formula should be selected for treatment:

A. Zuogui Pill B. Dabuyin Pill C. Zuogui Drink D. Zhibai Dihuang Pill E. Huqian Pill

Answer: E

13. 2021U3-2A1 Among impediment syndromes affecting organs chronically, the most common organ impediment is:

A. Lung impediment B. Heart impediment C. Liver impediment D. Kidney impediment E. Spleen impediment

Answer: B

14. 2021U3-16A1 The basic pathogenesis of tremor syndrome is:

A. Sinew vessel losing nourishment and spasm B. Qi mechanism counterflow disorder, ascent-descent dysfunction C. Pathogen qi impediment, meridian blockage D. Liver wind internal movement, sinew vessel losing nourishment E. Improper taxation, sinew vessel damage

Answer: D

15. 2024U3-17A1 The disease location of tremor syndrome is:

A. Liver-kidney B. Meridians C. Joints D. Sinews and vessels E. Muscles

Answer: D

16. 2001U3-24A1 The key in lumbar pain pathogenesis is:

A. Cold-dampness B. Dampness-heat C. Kidney deficiency D. Qi stagnation E. Blood stasis

Answer: C
