# Supplementary material for: Digitally Assisted Clinical Decision-Making in Traditional Chinese Medicine: Comparative Study of 5 Large Language Models
Source: JMIR Form Res. 2026 Mar 2;10:e80167. doi: 10.2196/80167 (PMC12954686; doi:10.2196/80167)
Supplement: Multimedia Appendix 2 [file formative-v10-e80167-s002.doc]

## Multimedia Appendix 2: Detailed Content of 30 Case Analyses

### Brief Case Analysis Questions (15 cases)

#### Case 1

【Case Summary】

Patient Ji, male, 48 years old. Initial consultation on February 14, 2019.

The patient has a family history of diabetes mellitus and usually prefers spicy food and rich, greasy diet. In the recent two months, he developed polydipsia, polyphagia, polyuria, and fatigue, with polyphagia being particularly prominent. Current symptoms: polyphagia with easy hunger, thirst, polyuria, weight loss, dry stool, yellow coating, and strong slippery pulse.

【Answer Requirements】

(1) Based on the above case summary, please provide: TCM disease diagnosis, TCM syndrome diagnosis, TCM diagnostic basis (including etiology and pathogenesis analysis), TCM treatment principles, formula, drug composition, dosage and decoction method.

(2) TCM disease differentiation: Please differentiate from goiter disease.

【TCM Disease Diagnosis】Diabetes mellitus (3 points)

【TCM Syndrome Diagnosis】Middle burner - Stomach heat exuberance syndrome (3 points)

【TCM Diagnostic Basis】

The patient presents with "polydipsia, polyphagia, polyuria, fatigue" as main symptoms, thus can be diagnosed as diabetes mellitus (1 point); based on "polyphagia with easy hunger, thirst, polyuria, weight loss, dry stool, yellow coating, strong slippery pulse," it can be diagnosed as middle burner stomach heat exuberance syndrome (1 point). This is caused by stomach fire internal blazing, stomach heat consuming grain, and depleting body fluids (2 points).

【TCM Disease Differentiation】

Both diabetes mellitus and goiter disease can present with polyphagia and weight loss symptoms. Goiter disease with qi constraint transforming fire and yin deficiency fire hyperactivity types presents with emotional excitement, polyphagia with easy hunger, progressive weight loss, palpitation, eye protrusion, and unilateral or bilateral neck swelling as characteristics. The polyphagia and weight loss are similar to middle burner of diabetes mellitus, but eye protrusion and anterior neck goiter distinguish it from diabetes mellitus, and it lacks the polydipsia and polyuria symptoms of diabetes mellitus. (3 points)

【TCM Treatment Principle】Clear stomach and drain fire, nourish yin and increase fluids (2 points)

【Formula Name】Modified Yunü Decoction (2 points)

【Drug Composition, Dosage and Decoction Method】(3 points)

Raw Gypsum (decoct first) 12g, Anemarrhena 6g, Coptis 6g, Gardenia 9g

Scrophularia 6g, Raw Rehmannia 15g, Ophiopogon 6g, Achyranthes 6g

3 doses, decocted in water. One dose daily, taken morning and evening.

#### Case 2

【Case Summary】

Patient Zhao, female, 45 years old, teacher, initial consultation October 2019.

Patient has had recurrent stomach pain for 3 years, and stomach pain attack triggered by anger 5 days ago. Symptoms: gastric distension and pain radiating to both hypochondria, pain relieved after belching, chest tightness with belching, constipation, thin white tongue coating, wiry pulse.

【Answer Requirements】

(1) Based on the above case summary, please provide: TCM disease diagnosis, TCM syndrome diagnosis, TCM diagnostic basis (including etiology and pathogenesis analysis), TCM treatment principles, formula, drug composition, dosage and decoction method.

(2) TCM disease differentiation: Please differentiate from abdominal pain.

【TCM Disease Diagnosis】Stomach pain (3 points)

【TCM Syndrome Diagnosis】Liver qi attacking stomach syndrome (3 points)

【TCM Diagnostic Basis】

The patient presents with "gastric distension and pain" as main symptom, thus can be diagnosed as stomach pain (1 point); based on "pain radiating to both hypochondria, pain relieved after belching, chest tightness with belching, constipation, thin white tongue coating, wiry pulse," it can be diagnosed as liver qi attacking stomach syndrome (1 point). This is caused by liver qi stagnation, transversely attacking stomach, stomach qi obstruction (2 points).

【TCM Disease Differentiation】

Abdominal pain presents with pain below the gastric region and above the pubic hairline as main symptom, while stomach pain presents with pain in the upper abdominal gastric region near the heart socket as main symptom. The two can be distinguished by pain location alone. However, since the stomach is located in the abdomen and connected to intestines, stomach pain can affect the abdomen, and abdominal pain can also involve the stomach, so differentiation should be based on the main pain location and how the disease starts. (3 points)

【TCM Treatment Principle】Soothe liver and regulate qi, harmonize stomach and stop pain (2 points)

【Formula Name】Modified Chaihu Shugan Powder (2 points)

【Drug Composition, Dosage and Decoction Method】(3 points)

Bupleurum 15g, Peony 9g, Chuanxiong 9g, Bitter Orange 12g

Tangerine Peel 9g, Licorice 6g, Cyperus 9g

3 doses, decocted in water. One dose daily, taken morning and evening.

#### **Case 3**

【Case Summary】

Patient Meng, male, 31 years old. Initial consultation July 14, 2021.

Patient started experiencing frequent short astringent urination, burning stabbing pain, reddish-yellow urine, lower abdominal urgency and distension from yesterday. Accompanied by bitter taste, nausea and vomiting, constipation, yellow greasy coating, slippery rapid pulse.

【Answer Requirements】

(1) Based on the above case summary, please provide: TCM disease diagnosis, TCM syndrome diagnosis, TCM diagnostic basis (including etiology and pathogenesis analysis), TCM treatment principles, formula, drug composition, dosage and decoction method.

(2) TCM disease differentiation: Please differentiate from urinary retention.

【TCM Disease Diagnosis】Strangury syndrome (3 points)

【TCM Syndrome Diagnosis】Heat strangury (3 points)

【TCM Diagnostic Basis】

The patient presents with "frequent astringent painful urination, lower abdominal urgency and distension" as main symptoms, thus can be diagnosed as strangury syndrome (1 point); based on "frequent short astringent urination, burning stabbing pain, reddish-yellow urine, lower abdominal urgency and distension, bitter taste, nausea and vomiting, constipation, yellow greasy coating, slippery rapid pulse," it can be diagnosed as heat strangury (1 point). This is caused by dampness-heat accumulating in lower burner, bladder qi transformation dysfunction (2 points).

【TCM Disease Differentiation】

Both strangury syndrome and urinary retention have symptoms of reduced urine volume and difficult urination, but strangury syndrome has frequent and painful urination with normal daily total urine volume, while urinary retention has no urinary pain and daily urine volume less than normal, severely even anuria. (3 points)

【TCM Treatment Principle】Clear heat, promote diuresis and unblock strangury (2 points)

【Formula Name】Modified Bazheng Powder (2 points)

【Drug Composition, Dosage and Decoction Method】(3 points)

Dianthus 10g, Polygonum aviculare 10g, Dioscorea 10g

Plantain Seed (wrapped) 10g, Rhubarb 10g, Phellodendron 9g

Talc (wrapped) 10g, Dandelion 10g, Viola 9g

3 doses, decocted in water. One dose daily, taken morning and evening.

#### **Case 4**

【Case Summary】

Patient Zhao, male, 66 years old. Initial consultation December 30, 2019.

Patient usually irritable and easily angered, recently over a month often unable to sleep with many dreams, even sleepless all night. Accompanied by dizziness and head distension, red eyes with tinnitus, dry bitter mouth, no desire for food, constipation with reddish urine, red tongue with yellow coating, wiry rapid pulse.

【Answer Requirements】

(1) Based on the above case summary, please provide: TCM disease diagnosis, TCM syndrome diagnosis, TCM diagnostic basis (including etiology and pathogenesis analysis), TCM treatment principles, formula, drug composition, dosage and decoction method.

(2) TCM disease differentiation: Please differentiate from temporary insomnia.

【TCM Disease Diagnosis】Insomnia (3 points)

【TCM Syndrome Diagnosis】Liver fire disturbing heart syndrome (3 points)

【TCM Diagnostic Basis】

The patient presents with "insomnia with many dreams, even sleepless all night" as main symptom, thus can be diagnosed as insomnia (1 point); based on "insomnia with many dreams, even sleepless all night, irritable and easily angered, dizziness and head distension, red eyes with tinnitus, dry bitter mouth, no desire for food, constipation with reddish urine, red tongue with yellow coating, wiry rapid pulse," it can be diagnosed as liver fire disturbing heart syndrome (1 point). This is caused by liver constraint transforming fire, ascending to disturb heart spirit (2 points).

【TCM Disease Differentiation】

Insomnia refers simply to insomnia as main symptom, presenting as persistent, severe sleep difficulties. Temporary insomnia caused by emotional influence or environmental changes does not belong to pathological conditions. (3 points)

【TCM Treatment Principle】Soothe liver and drain fire, calm heart and settle spirit (2 points)

【Formula Name】Modified Longdan Xiegan Decoction (2 points)

【Drug Composition, Dosage and Decoction Method】(3 points)

Gentian 6g, Scutellaria 9g, Gardenia 9g, Plantain Seed (wrapped) 9g

Alisma 12g, Angelica 3g, Raw Rehmannia 9g, Raw Dragon Bone (decoct first) 6g

Magnetite (decoct first) 6g, Licorice 6g, Bupleurum 6g, Raw Oyster Shell (decoct first) 6g

3 doses, decocted in water. One dose daily, taken morning and evening.

#### **Case 5**

【Case Summary】

Patient Zhu, female, 48 years old, married, office worker. Initial consultation February 23, 2016.

Patient went out yesterday without paying attention to keeping warm, developed headache after returning home. Current symptoms: headache radiating to neck and back with tight constricting sensation, aversion to wind and fear of cold, headache worse with wind exposure, no thirst, thin white coating, floating tight pulse.

【Answer Requirements】

(1) Based on the above case summary, please provide: TCM disease diagnosis, TCM syndrome diagnosis, TCM diagnostic basis (including etiology and pathogenesis analysis), TCM treatment principles, formula, drug composition, dosage and decoction method.

(2) TCM disease differentiation: Please differentiate from vertigo.

【TCM Disease Diagnosis】Headache (3 points)

【TCM Syndrome Diagnosis】Wind-cold headache (3 points)

【TCM Diagnostic Basis】

The patient presents with "headache" as main symptom, thus can be diagnosed as headache (1 point); based on "headache radiating to neck and back with tight constricting sensation, aversion to wind and fear of cold, headache worse with wind exposure, no thirst, thin white coating, floating tight pulse," it can be diagnosed as wind-cold headache (1 point). This is caused by wind-cold external invasion, ascending to attack vertex, congealing and stagnating meridians (2 points).

【TCM Disease Differentiation】

Headache and vertigo can appear separately or simultaneously. Comparing the two, headache causes include both external and internal injury aspects, while vertigo is mainly internal injury. In clinical manifestations, headache presents mainly with pain and more excess patterns; while vertigo presents mainly with dizziness and more deficiency patterns. (3 points)

【TCM Treatment Principle】Disperse wind, scatter cold and stop pain (2 points)

【Formula Name】Modified Chuanxiong Chatiao Powder (2 points)

【Drug Composition, Dosage and Decoction Method】(3 points)

Chuanxiong 12g, Schizonepeta 12g, Saposhnikovia 6g, Cicada Shell 9g

Sophora root 6g, Xanthium 6g, Angelica dahurica 9g, Ligusticum 9g

Angelica 9g, Prepared Rehmannia 9g, Licorice 6g

3 doses, decocted in water. One dose daily, taken morning and evening.

#### **Case 6**

【Case Summary】

Patient Yan, male, 46 years old, cadre. Initial consultation July 20, 2018.

Patient has had loose stools for over 1 year, condition varies in severity, often aggravated by depression and anger. Current symptoms: diarrhea with wandering abdominal pain, loose stools 3 times daily, accompanied by fatigue, chest and hypochondriac distension, belching with poor appetite, epigastric and abdominal distension, pale red tongue, thin white coating, wiry pulse.

【Answer Requirements】

(1) Based on the above case summary, please provide: TCM disease diagnosis, TCM syndrome diagnosis, TCM diagnostic basis (including etiology and pathogenesis analysis), TCM treatment principles, formula, drug composition, dosage and decoction method.

(2) TCM disease differentiation: Please differentiate from dysentery.

【TCM Disease Diagnosis】Diarrhea (3 points)

【TCM Syndrome Diagnosis】Liver qi overwhelming spleen syndrome (3 points)

【TCM Diagnostic Basis】

The patient presents with "diarrhea with wandering abdominal pain, loose stools" as main symptoms, thus can be diagnosed as diarrhea (1 point); based on "fatigue, chest and hypochondriac distension, belching with poor appetite, epigastric and abdominal distension, pale red tongue, thin white coating, wiry pulse," it can be diagnosed as liver qi overwhelming spleen syndrome (1 point). This is caused by liver qi discomfort, transversely attacking spleen, spleen losing healthy transportation (2 points).

【TCM Disease Differentiation】

Both are diseases with increased bowel movement frequency and thin stool quality. Diarrhea presents with increased bowel movement frequency, thin loose stools, even watery, or undigested food, without pus and blood in stool, and no tenesmus or minimal abdominal pain. Dysentery is characterized by abdominal pain, tenesmus, and red-white purulent bloody stools. (3 points)

【TCM Treatment Principle】Suppress liver and support spleen (2 points)

【Formula Name】Modified Tongxie Yaofang (2 points)

【Drug Composition, Dosage and Decoction Method】(3 points)

Tangerine Peel 10g, White Atractylodes 15g, Peony 12g, Saposhnikovia 9g

3 doses, decocted in water. One dose daily, taken morning and evening.

#### **Case 7**

【Case Summary】

Patient Ma, female, 65 years old, farmer. Initial consultation March 6, 2021.

Patient often worked by the seaside when young, has had lumbar pain for nearly 40 years, cold heavy pain in lumbar region, difficult turning and movement, gradually worsening, pain not reduced when lying still, aggravated by cold and rainy weather, pale tongue, white greasy coating, deep slow pulse.

【Answer Requirements】

(1) Based on the above case summary, please provide: TCM disease diagnosis, TCM syndrome diagnosis, TCM diagnostic basis (including etiology and pathogenesis analysis), TCM treatment principles, formula, drug composition, dosage and decoction method.

(2) TCM disease differentiation: Please differentiate from kidney impediment.

【TCM Disease Diagnosis】Lumbar pain (3 points)

【TCM Syndrome Diagnosis】Cold-dampness lumbar pain (3 points)

【TCM Diagnostic Basis】

The patient presents with "lumbar pain" as main symptom, thus can be diagnosed as lumbar pain (1 point); based on "cold heavy pain in lumbar region, difficult turning and movement, gradually worsening, pain not reduced when lying still, aggravated by cold and rainy weather, pale tongue, white greasy coating, deep slow pulse," it can be diagnosed as cold-dampness lumbar pain (1 point). This is caused by cold-dampness blocking, obstructing qi-blood, meridians unfavorable (2 points).

【TCM Disease Differentiation】

Lumbar pain presents with lumbar pain as main symptom; kidney impediment refers to lumbar back stiffness and curvature, inability to flex and extend, difficult movement, mostly developing from chronic bone impediment. (3 points)

【TCM Treatment Principle】Scatter cold and move dampness, warm meridians and unblock collaterals (2 points)

【Formula Name】Modified Ganjiang Lingzhu Decoction (2 points)

【Drug Composition, Dosage and Decoction Method】(3 points)

Dried Ginger 10g, Cinnamon Twig 15g, Poria 15g, Achyranthes 12g

White Atractylodes 12g, Eucommia 15g, Loranthus 12g, Dipsacus 15g

Asarum 3g, Licorice 5g

3 doses, decocted in water. One dose daily, taken morning and evening.

#### **Case 8**

【Case Summary】

Patient Fang, male, 43 years old, married, worker. Initial consultation September 29, 2015.

Patient traveled for business 2 days ago, developed dry cough the next day, continuous choking cough, throat itching, dry throat pain, dry lips and nose, scanty sticky sputum difficult to expectorate, dry mouth, with aversion to wind, fever, red dry tongue with scanty fluid, thin white coating, floating rapid pulse.

【Answer Requirements】

(1) Based on the above case summary, please provide: TCM disease diagnosis, TCM syndrome diagnosis, TCM diagnostic basis (including etiology and pathogenesis analysis), TCM treatment principles, formula, drug composition, dosage and decoction method.

(2) TCM disease differentiation: Please differentiate from lung consumption.

【TCM Disease Diagnosis】Cough (3 points)

【TCM Syndrome Diagnosis】Wind-dryness injuring lung syndrome (3 points)

【TCM Diagnostic Basis】

The patient presents with "cough" as main symptom, thus can be diagnosed as cough (1 point); based on "dry cough, continuous choking cough, throat itching, dry throat pain, dry lips and nose, scanty sticky sputum difficult to expectorate, dry mouth, with aversion to wind, fever, red dry tongue with scanty fluid, thin white coating, floating rapid pulse," it can be diagnosed as wind-dryness injuring lung syndrome (1 point). This is caused by wind-dryness injuring lung, lung losing clear moistening (2 points).

【TCM Disease Differentiation】

Both cough and lung consumption can have cough and sputum symptoms, but lung consumption is caused by "consumption insect" infection, is contagious, and accompanied by tidal fever, night sweats, hemoptysis, weight loss, which can differentiate it. (3 points)

【TCM Treatment Principle】Disperse wind and clear lung, moisten dryness and stop cough (2 points)

【Formula Name】Modified Sangxing Decoction (2 points)

【Drug Composition, Dosage and Decoction Method】(3 points)

Mulberry Leaf 6g, Apricot Kernel 9g, Fritillaria 3g, Adenophora 6g

Tangerine Peel 6g, Light Soybean 6g, Pear Peel 6g, Arctium 3g

3 doses, decocted in water. One dose daily, taken morning and evening.

#### **Case 9**

【Case Summary】

Patient Shou, male, 29 years old, married, office worker. Initial consultation May 15, 2018.

Patient has had right hypochondriac distending pain for 1 year, wandering and unfixed, varies in severity, related to emotions, chest tightness with abdominal distension, attacks of sighing, thin white tongue coating, wiry pulse.

【Answer Requirements】

(1) Based on the above case summary, please provide: TCM disease diagnosis, TCM syndrome diagnosis, TCM diagnostic basis (including etiology and pathogenesis analysis), TCM treatment principles, formula, drug composition, dosage and decoction method.

(2) TCM disease differentiation: Please differentiate from gastric pain.

【TCM Disease Diagnosis】Hypochondriac pain (3 points)

【TCM Syndrome Diagnosis】Liver qi stagnation syndrome (3 points)

【TCM Diagnostic Basis】

The patient presents with "right hypochondriac distending pain for 1 year" as main symptom, thus can be diagnosed as hypochondriac pain (1 point); based on "wandering and unfixed, varies in severity, related to emotions, chest tightness with abdominal distension, attacks of sighing, thin white coating, wiry pulse," it can be diagnosed as liver qi stagnation syndrome (1 point). This is caused by liver losing free coursing, qi mechanism stagnant, collateral vessels disharmony (2 points).

【TCM Disease Differentiation】

Both diseases share common liver constraint pathogenesis. But disease locations differ - gastric pain is located in gastric region with frequent belching, acid swallowing, noisy stomach and other stomach descent dysfunction symptoms. Hypochondriac pain is located in hypochondriac regions with dizziness, bitter mouth, chest tightness, sighing symptoms. (3 points)

【TCM Treatment Principle】Soothe liver and regulate qi (2 points)

【Formula Name】Modified Chaihu Shugan Powder (2 points)

【Drug Composition, Dosage and Decoction Method】(3 points)

White Peony 9g, Bupleurum 12g, Melia 9g, Bitter Orange 9g

Curcuma 9g, Licorice 3g, Cyperus 9g, Chuanxiong 9g

3 doses, decocted in water. One dose daily, taken morning and evening.

#### **Case 10**

【Case Summary】

Patient Li, male, 41 years old, married, cadre. Initial consultation August 8, 2016.

Patient developed fever, aversion to cold, sneezing, headache, nasal congestion, runny nose 2 days ago. Took cold medicine at home with poor effect. Current symptoms: fever, slight aversion to wind, unsatisfactory sweating, sweating but fever not relieving, nasal congestion, yellow turbid nasal discharge, red tongue tip and border, thin white slightly yellow coating, floating rapid pulse.

【Answer Requirements】

(1) Based on the above case summary, please provide: TCM disease diagnosis, TCM syndrome diagnosis, TCM diagnostic basis (including etiology and pathogenesis analysis), TCM treatment principles, formula, drug composition, dosage and decoction method.

(2) TCM disease differentiation: Please differentiate from seasonal influenza.

【TCM Disease Diagnosis】Common cold (3 points)

【TCM Syndrome Diagnosis】Wind-heat cold (3 points)

【TCM Diagnostic Basis】

The patient presents with "fever, aversion to cold, sneezing, headache, nasal congestion, runny nose" as main symptoms, thus can be diagnosed as common cold (1 point); based on "fever, slight aversion to wind, unsatisfactory sweating, sweating but fever not relieving, nasal congestion, yellow turbid nasal discharge, red tongue tip and border, thin white slightly yellow coating, floating rapid pulse," it can be diagnosed as wind-heat cold (1 point). This is caused by wind-heat attacking exterior, heat constraint in skin, defensive exterior disharmony, lung losing clear descent (2 points).

【TCM Disease Differentiation】

Common cold has mild condition, systemic symptoms not severe, little transformation. During climate changes, incidence can increase, but no obvious epidemic characteristics. If cold persists over 1 week, fever not relieving or worsening, should consider cold complicating with other diseases, transformation inward. Seasonal influenza has severe condition, acute onset, prominent systemic symptoms, can have transformation, heat entering interior, secondary or combined other diseases, with extensive contagiousness and epidemic nature. (3 points)

【TCM Treatment Principle】Acrid-cool resolving exterior (2 points)

【Formula Name】Yinqiao Powder or Congchi Jiegeng Decoction modified (2 points)

【Drug Composition, Dosage and Decoction Method】(3 points)

Honeysuckle 12g, Forsythia 12g, Bamboo Leaf 9g, Schizonepeta 6g

Arctium 9g, Light Soybean 6g, Mentha 9g, Licorice 6g

Platycodon 9g, Reed Root 12g

3 doses, decocted in water. One dose daily, taken morning and evening.

#### **Case 11**

【Case Summary】

Patient Zhao, female, 28 years old. Initial consultation January 6, 2019.

Patient returned from out of town overnight yesterday, felt cold discomfort. Current symptoms: wheezing sound like water fowl in throat, rapid breathing, dyspnea with qi rebellion, chest and diaphragm fullness like obstruction, mild cough, scanty sputum difficult to expectorate, white with many bubbles, aversion to cold with cold body, blue-gray complexion, white slippery tongue coating, floating tight pulse.

【Answer Requirements】

(1) Based on the above case summary, please provide: TCM disease diagnosis, TCM syndrome diagnosis, TCM diagnostic basis (including etiology and pathogenesis analysis), TCM treatment principles, formula, drug composition, dosage and decoction method.

(2) TCM disease differentiation: Please differentiate from dyspnea syndrome.

【TCM Disease Diagnosis】Asthma disease (3 points)

【TCM Syndrome Diagnosis】Cold asthma syndrome (3 points)

【TCM Diagnostic Basis】

The patient presents with "wheezing sound like water fowl in throat" as main symptom, thus can be diagnosed as asthma disease (1 point); based on "rapid breathing, dyspnea with qi rebellion, chest and diaphragm fullness like obstruction, mild cough, scanty sputum difficult to expectorate, white with many bubbles, aversion to cold with cold body, blue-gray complexion, white slippery tongue coating, floating tight pulse," it can be diagnosed as cold asthma syndrome (1 point). This is caused by cold phlegm latent in lung, triggered by exposure, phlegm ascending qi obstruction, lung losing diffusion (2 points).

【TCM Disease Differentiation】

Both asthma disease and dyspnea syndrome have rapid breathing and difficulty manifestations. Asthma must include dyspnea, but dyspnea not necessarily includes asthma. Asthma refers to sound, with wheezing sound in throat, is a repeatedly attacking independent disease; dyspnea refers to breathing, as breathing difficulty, is a symptom of various acute and chronic lung system diseases. (3 points)

【TCM Treatment Principle】Diffuse lung and scatter cold, transform phlegm and calm dyspnea (2 points)

【Formula Name】Shegan Mahuang Decoction or Xiaoqinglong Decoction modified (2 points)

【Drug Composition, Dosage and Decoction Method】(3 points)

Ephedra 9g, Belamcanda 9g, Dried Ginger 6g, Asarum 6g

Pinellia 9g, Aster 6g, Coltsfoot 6g, Schisandra 3g

Jujube 6g, Licorice 6g

3 doses, decocted in water. One dose daily, taken morning and evening.

#### **Case 12**

【Case Summary】

Patient Tian, male, 40 years old, cadre. Hospitalized November 11, 2018.

Patient had aversion to wind and fever for 5 days, now feels joint pain and soreness, inflexible flexion-extension, pain wandering between bilateral knees, elbows and ankles, with left knee and ankle joints more severe, thin white tongue coating, floating moderate pulse.

【Answer Requirements】

(1) Based on the above case summary, please provide: TCM disease diagnosis, TCM syndrome diagnosis, TCM diagnostic basis (including etiology and pathogenesis analysis), TCM treatment principles, formula, drug composition, dosage and decoction method.

(2) TCM disease differentiation: Please differentiate from atrophy syndrome.

【TCM Disease Diagnosis】Impediment syndrome (3 points)

【TCM Syndrome Diagnosis】Wind-cold-dampness impediment - wandering impediment (3 points)

【TCM Diagnostic Basis】

The patient presents with "joint pain and soreness, inflexible flexion-extension" as main symptoms, thus can be diagnosed as impediment syndrome (1 point); based on "pain wandering between bilateral knees, elbows and ankles, with left knee and ankle joints more severe, thin white coating, floating moderate pulse," it can be diagnosed as wandering impediment (1 point). This is caused by wind pathogen combining cold-dampness, staying in meridians, blocking qi-blood (2 points).

【TCM Disease Differentiation】

Impediment syndrome is caused by wind, cold, dampness, heat pathogens flowing into meridians, blocking and obstructing. Differential points first lie in pain vs. no pain - impediment syndrome presents mainly with joint pain, while atrophy syndrome presents with limb weakness without pain symptoms; second, observe limb movement disorders - atrophy syndrome is powerless movement, impediment syndrome is pain affecting movement; furthermore, some atrophy syndrome cases have muscle atrophy from disease onset, while impediment syndrome has muscle atrophy from long-term disuse due to severe pain or joint stiffness unable to move. (3 points)

【TCM Treatment Principle】Dispel wind and unblock collaterals, scatter cold and eliminate dampness (2 points)

【Formula Name】Modified Fangfeng Decoction (2 points)

【Drug Composition, Dosage and Decoction Method】(3 points)

Saposhnikovia 12g, Ephedra 6g, Cinnamon Twig 9g, Pueraria 15g

Angelica 12g, Poria 12g, Licorice 6g, Fresh Ginger 3g

3 doses, decocted in water. One dose daily, taken morning and evening.

#### **Case 13**

【Case Summary】

Patient, male, 15 years old, student. Initial consultation January 10, 2022.

Patient has had picky eating habits since childhood, frequently irregular diet, often felt dizzy, mental fatigue, palpitation and shortness of breath, limb fatigue, many dreams, restless sleep for 2 years. Dizziness worsened in recent week while reviewing for exams, came for consultation. Thin build, pale complexion, pale lips and nails, heart and lungs normal, soft flat abdomen without tenderness, normal bowel and urinary function, pale red tongue, thin white coating, fine weak pulse.

【Answer Requirements】

(1) Based on the above case summary, please provide: TCM disease diagnosis, TCM syndrome diagnosis, TCM diagnostic basis (including etiology and pathogenesis analysis), TCM treatment principles, formula, drug composition, dosage and decoction method.

(2) TCM disease differentiation: Please differentiate from stroke.

【TCM Disease Diagnosis】Vertigo (3 points)

【TCM Syndrome Diagnosis】Qi-blood deficiency syndrome (3 points)

【TCM Diagnostic Basis】

The patient presents with "dizziness" as main symptom, thus can be diagnosed as vertigo (1 point); based on "dizziness, mental fatigue, palpitation and shortness of breath, limb fatigue, many dreams, restless sleep, thin build, pale complexion, pale lips and nails, normal bowel and urinary function, pale red tongue, thin white coating, fine weak pulse," it can be diagnosed as qi-blood deficiency syndrome (1 point). This is caused by qi-blood deficiency, clear yang not extending, brain losing nourishment (2 points).

【TCM Disease Differentiation】

Stroke is characterized by sudden collapse, unconsciousness, mouth and tongue deviation, hemiplegia, aphasia, or without collapse but only facial paralysis and limb disability. Stroke collapse resembles severe vertigo cases - severe vertigo can also cause collapse, fainting has memory blank, instant awakening, but without hemiplegia, unconsciousness, mouth and tongue deviation symptoms. Some stroke patients also have vertigo and headache as prodromal manifestations, so clinical attention should be paid to differences and connections between stroke and vertigo. (3 points)

【TCM Treatment Principle】Supplement qi and blood, regulate and nourish heart-spleen (2 points)

【Formula Name】Modified Guipi Decoction (2 points)

【Drug Composition, Dosage and Decoction Method】(3 points)

Astragalus 12g, White Atractylodes 10g, Poria 12g, Angelica 9g

Chuanxiong 9g, Jujube Seed 9g, Aucklandia 6g, Albizzia Flower 12g

Drynaria 10g, Prepared Rehmannia 10g, Hawthorn 6g, Licorice 6g

3 doses, decocted in water. One dose daily, taken morning and evening.

#### **Case 14**

【Case Summary】

Patient Ren, female, 41 years old, married, office worker. Initial consultation October 17, 2019.

Patient usually has weak constitution, developed low fever 3 months ago, fatigue. Current symptoms: fever with low heat degree, often triggered or aggravated by fatigue, lassitude and fatigue, shortness of breath with lazy speech, spontaneous sweating, susceptible to colds, poor appetite with loose stools, pale tongue, thin white coating, fine weak pulse.

【Answer Requirements】

(1) Based on the above case summary, please provide: TCM disease diagnosis, TCM syndrome diagnosis, TCM diagnostic basis (including etiology and pathogenesis analysis), TCM treatment principles, formula, drug composition, dosage and decoction method.

(2) TCM disease differentiation: Please differentiate from external pathogenic fever.

【TCM Disease Diagnosis】Internal injury fever (3 points)

【TCM Syndrome Diagnosis】Qi deficiency fever syndrome (3 points)

【TCM Diagnostic Basis】

The patient presents with "low fever for 3 months" as main symptom, thus can be diagnosed as internal injury fever (1 point); based on "often triggered or aggravated by fatigue, lassitude and fatigue, shortness of breath with lazy speech, spontaneous sweating, susceptible to colds, poor appetite with loose stools, pale tongue, thin white coating, fine weak pulse," it can be diagnosed as qi deficiency fever syndrome (1 point). This is caused by middle qi insufficiency, yin fire internally generated (2 points).

【TCM Disease Differentiation】

Internal injury fever has slow onset, long course, mostly low fever, or subjective fever sensation without actual temperature elevation, few high fever cases. No aversion to cold, or although there is cold sensation, it is relieved by clothing. Often accompanied by dizziness, mental fatigue, spontaneous sweating, night sweats, weak pulse. No head-body pain, nasal congestion, runny nose, floating pulse caused by external pathogen invasion. External pathogenic fever characteristics are: caused by external pathogen invasion, acute onset, short course, fever initially mostly accompanied by aversion to cold, aversion to cold not relieved by clothing. Fever degree mostly higher, fever types vary with different diseases. Initially often accompanied by head-body pain, nasal congestion, runny nose, cough, floating pulse and other exterior symptoms. External pathogenic fever is caused by external pathogen invasion, healthy and pathogenic struggling, mostly excess patterns. (3 points)

【TCM Treatment Principle】Benefit qi and strengthen spleen, sweet-warm removing heat (2 points)

【Formula Name】Modified Buzhong Yiqi Decoction (2 points)

【Drug Composition, Dosage and Decoction Method】(3 points)

Astragalus 15g, White Atractylodes 9g, Tangerine Peel 6g, Cimicifuga 6g

Bupleurum 6g, Codonopsis 6g, Licorice 9g, Angelica 3g

3 doses, decocted in water. One dose daily, taken morning and evening.

#### **Case 15**

【Case Summary】

Patient Qi, male, 24 years old. Initial consultation June 19, 2019.

Patient usually has irregular diet, fond of spicy food, had drinking feast with friends last night. This morning presents with: epigastric and abdominal distension and fullness, pain refusing pressure, belching rotten and swallowing acid, aversion to food with nausea and vomiting, constipation, thick greasy tongue coating, slippery pulse.

【Answer Requirements】

(1) Based on the above case summary, please provide: TCM disease diagnosis, TCM syndrome diagnosis, TCM diagnostic basis (including etiology and pathogenesis analysis), TCM treatment principles, formula, drug composition, dosage and decoction method.

(2) TCM disease differentiation: Please differentiate from gastric pain.

【TCM Disease Diagnosis】Abdominal pain (3 points)

【TCM Syndrome Diagnosis】Food accumulation stagnation syndrome (3 points)

【TCM Diagnostic Basis】

The patient presents with "epigastric and abdominal distension and fullness, pain refusing pressure" as main symptoms, thus can be diagnosed as abdominal pain (1 point); based on "belching rotten and swallowing acid, aversion to food with nausea and vomiting, constipation, thick greasy tongue coating, slippery pulse," it can be diagnosed as food accumulation stagnation syndrome (1 point). This is caused by food stagnation internally stopping, transportation dysfunction, stomach-intestine disharmony (2 points).

【TCM Disease Differentiation】

The stomach is located in the abdomen and connected to intestines, so abdominal pain often accompanies gastric pain symptoms, and gastric pain may also have abdominal pain manifestations, requiring differentiation. First is location difference - gastric pain is in the subxiphoid gastric region, abdominal pain is below gastric region above pubic hairline; second is accompanying symptoms difference - gastric pain often accompanies nausea, belching and other gastric disease symptoms, abdominal pain can accompany constipation, diarrhea or urinary frequency, urgency symptoms. (3 points)

【TCM Treatment Principle】Digest food and guide stagnation, regulate qi and stop pain (2 points)

【Formula Name】Modified Zhishi Daozhiwan (2 points)

【Drug Composition, Dosage and Decoction Method】(3 points)

Bitter Orange 6g, Medicated Leaven 6g, Scutellaria 3g, Rhubarb (add later) 9g

Coptis 3g, Alisma 3g, White Atractylodes 3g, Poria 3g

3 doses, decocted in water. One dose daily, taken morning and evening.

### Complex Case Analysis Questions (15 cases)

#### **Case 1**

【Case Summary】

Patient He, male, 32 years old. Consultation March 18, 2021.

Chief complaint: Irritable and easily angered temperament, insomnia, headache with red eyes for 3 months.

History: Reports recent years of stock trading, due to unpredictable stock market changes and sharp price drops, mood disturbance, irritability and anger, unable to sleep at night, taking Western medicine sedatives can sleep for several hours, next day headache with red eyes, head burning heat. Long-term dry bitter mouth, tasteless food, constipation, chest tightness with hypochondriac distension. Sought TCM treatment on March 18.

Current symptoms: mood disturbance, irritability and anger, restless sleep, headache with red eyes, head burning heat, chest tightness with hypochondriac pain, occasional nausea and acid regurgitation, dry bitter mouth, poor appetite, constipation. Red tongue with yellow coating, wiry rapid pulse.

【Answer Requirements】

(1) Based on the above case summary, please provide: TCM disease diagnosis, TCM syndrome diagnosis, TCM diagnostic basis (including etiology and pathogenesis analysis), TCM treatment principles, formula, drug composition, dosage and decoction method.

(2) TCM disease differentiation: Please differentiate from visceral agitation.

【TCM Disease Diagnosis】Depression syndrome (3 points)

【TCM Syndrome Diagnosis】Qi constraint transforming fire syndrome (3 points)

【TCM Diagnostic Basis】

The patient presents with "mood disturbance, irritability and anger" as main symptoms, thus can be diagnosed as depression syndrome (1 point); based on "mood disturbance, irritability and anger, restless sleep, headache with red eyes, head burning heat, chest tightness with hypochondriac pain, occasional nausea and acid regurgitation, dry bitter mouth, poor appetite, constipation, red tongue with yellow coating, wiry rapid pulse," it can be diagnosed as qi constraint transforming fire syndrome (1 point). This is caused by liver constraint transforming fire, transversely attacking stomach (2 points).

【TCM Disease Differentiation】

Visceral agitation is heart spirit losing nourishment syndrome in depression syndrome, presenting with characteristics of sad desire to cry, frequent yawning, mostly occurring in young and middle-aged women, manifesting intermittently under mental stimulation, can be like normal people when not attacking. (3 points)

【TCM Treatment Principle】Soothe liver and resolve constraint, clear liver and drain fire (2 points)

【Formula Name】Modified Danzhi Xiaoyao Powder (2 points)

【Drug Composition, Dosage and Decoction Method】(3 points)

Gardenia 10g, Moutan 10g, Bupleurum 10g, Mentha 8g

Angelica 10g, White Peony 15g, Poria 15g, Evodia 3g

Melia 6g, Prunella 15g, Gentian 10g, Jujube Seed 10g

Rhubarb 6g, Bitter Orange 10g, Licorice 6g

3 doses, decocted in water. One dose daily, taken morning and evening.

#### **Case 2**

【Case Summary】

Patient, male, 78 years old. Consultation March 2, 2019.

Patient has history of hepatitis B for over 10 years. Two weeks ago developed abdominal distension without obvious cause and came to hospital. Current symptoms: large firm abdominal distension, epigastric and abdominal distension urgency, vexation heat with bitter mouth, thirst without desire to drink, yellow skin, scanty reddish urine, constipation, red tongue tip and border, yellow greasy coating, wiry rapid pulse.

【Answer Requirements】

(1) Based on the above case summary, please provide: TCM disease diagnosis, TCM syndrome diagnosis, TCM diagnostic basis (including etiology and pathogenesis analysis), TCM treatment principles, formula, drug composition, dosage and decoction method.

(2) TCM disease differentiation: Please differentiate from focal distension.

【TCM Disease Diagnosis】Drum distension (3 points)

【TCM Syndrome Diagnosis】Dampness-heat accumulation syndrome (3 points)

【TCM Diagnostic Basis】

The patient presents with "large abdominal distension" as main symptom, thus can be diagnosed as drum distension (1 point); based on "large firm abdominal distension, epigastric and abdominal distension urgency, vexation heat with bitter mouth, thirst without desire to drink, yellow skin, scanty reddish urine, constipation, red tongue tip and border, yellow greasy coating, wiry rapid pulse," it can be diagnosed as dampness-heat accumulation syndrome (1 point). This is caused by dampness-heat exuberance, accumulating in middle burner, turbid water internally stopping (2 points).

【TCM Disease Differentiation】

Both drum distension and focal distension have abdominal distension symptoms, but gastric focal distension is seen in upper abdomen with no visible distended appearance, soft on palpation; drum distension affects entire abdomen, gray-yellow skin color with visible vessel network, tight abdominal skin on palpation. (3 points)

【TCM Treatment Principle】Clear heat and promote diuresis, attack and drive out water (2 points)

【Formula Name】Zhongman Fenxiao Pill (2 points)

【Drug Composition, Dosage and Decoction Method】(3 points)

White Atractylodes 3g, Ginseng 3g, Honey-fried Licorice 3g, Polyporus 3g, Turmeric 3g, White Poria 6g

Dried Fresh Ginger 6g, Amomum 6g, Alisma 9g, Anemarrhena 12g, Scutellaria 36g, Coptis 15g

Pinellia 15g, Bitter Orange 15g, Magnolia 30g, Tangerine Peel 9g

3 doses, decocted in water. One dose daily, taken morning and evening.

#### **Case 3**

【Case Summary】

Patient Yu, male, 77 years old, married, retired. Consultation November 1, 2019.

Patient developed right upper abdominal pain 20 days ago without obvious cause, hospitalized with significantly elevated alpha-fetoprotein, CT examination suggested "liver cancer." Current symptoms: right hypochondriac pain, yellow body and eyes, dry bitter mouth, vexation and irritability, chest tightness with nausea, red tongue, yellow greasy coating, wiry slippery pulse.

【Answer Requirements】

(1) Based on the above case summary, please provide: TCM disease diagnosis, TCM syndrome diagnosis, TCM diagnostic basis (including etiology and pathogenesis analysis), TCM treatment principles, formula, drug composition, dosage and decoction method.

(2) TCM disease differentiation: Please differentiate from benign tumors.

【TCM Disease Diagnosis】Cancer disease (3 points)

【TCM Syndrome Diagnosis】Dampness-heat toxin constraint syndrome (3 points)

【TCM Diagnostic Basis】

Patient has right upper abdominal pain, examination suggests liver cancer diagnosis, thus can be diagnosed as cancer disease (1 point); based on "right hypochondriac pain, yellow body and eyes, dry bitter mouth, vexation and irritability, chest tightness with nausea, red tongue, yellow greasy coating, wiry slippery pulse," it can be diagnosed as dampness-heat toxin constraint syndrome (1 point). This is caused by dampness pathogen transforming heat, dampness-heat accumulating toxin (2 points).

【TCM Disease Differentiation】

Benign tumors grow slowly, skin unchanged, except sebaceous cysts, no adhesion to skin, smooth tumor surface, no adhesion to surroundings, clear borders, good mobility, generally soft texture, generally asymptomatic, large tumors or those in special locations can produce compression symptoms. Cancer disease grows rapidly, often adheres to skin, depressed or forming ulcers, rough tumor surface, no capsule, often adheres to surroundings or skin, poor mobility or fixed, hard texture, no elasticity, early symptoms hidden, can have unexplained weight loss, fever, bleeding, or corresponding symptoms of affected areas. (3 points)

【TCM Treatment Principle】Clear heat and promote diuresis, detoxify and scatter binding (2 points)

【Formula Name】Longdan Xiegan Decoction combined with Wuwei Xiaodu Drink modified (2 points)

【Drug Composition, Dosage and Decoction Method】(3 points)

Gentian 6g, Wild Chrysanthemum 9g, Scutellaria 9g, Gardenia 9g

Honeysuckle 15g, Akebia 6g, Rehmannia 9g, Alisma 12g

Raw Licorice 6g, Bupleurum 6g, Angelica 3g, Plantain Seed (wrapped) 9g

Dandelion 9g, Viola 9g, Begonia fimbristipula 9g

3 doses, decocted in water. One dose daily, taken morning and evening.

#### **Case 4**

【Case Summary】

Patient Zhao, male, 20 years old, college student. Initial consultation September 2018.

One week ago caught in rain, developed aversion to cold, fever, no sweating, generalized pain, sore throat, nasal congestion with runny nose, mild cough, self-administered Tylenol, temperature normalized, runny nose reduced, generalized pain relieved, but cough gradually worsened, self-administered licorice tablets and roxithromycin ineffectively. Currently severe cough, coarse rapid breathing, profuse yellow sticky thick sputum, chest and hypochondriac distension, pain when coughing, flushed face, dry sticky mouth, red tongue, thin yellow greasy coating, slippery rapid pulse.

【Answer Requirements】

(1) Based on the above case summary, please provide: TCM disease diagnosis, TCM syndrome diagnosis, TCM diagnostic basis (including etiology and pathogenesis analysis), TCM treatment principles, formula, drug composition, dosage and decoction method.

(2) TCM disease differentiation: Please differentiate from dyspnea syndrome.

【TCM Disease Diagnosis】Cough (3 points)

【TCM Syndrome Diagnosis】Phlegm-heat constraining lung syndrome (3 points)

【TCM Diagnostic Basis】

The patient presents with "cough" as main symptom, thus can be diagnosed as cough (1 point); based on "severe cough, coarse rapid breathing, profuse yellow sticky thick sputum, chest and hypochondriac distension, pain when coughing, flushed face, dry sticky mouth, red tongue, thin yellow greasy coating, slippery rapid pulse," it can be diagnosed as phlegm-heat constraining lung syndrome (1 point). This is caused by phlegm-heat blocking lung, lung losing descent (2 points).

【TCM Disease Differentiation】

Both cough and dyspnea syndrome belong to lung qi ascending rebellion diseases, clinically often see cough and dyspnea together, but cough presents mainly with qi rebellion sound and expectoration as main symptom, dyspnea syndrome presents with breathing difficulty, even inability to lie flat as clinical characteristics. (3 points)

【TCM Treatment Principle】Clear heat and descend lung, open phlegm and stop cough (2 points)

【Formula Name】Modified Qingjin Huatan Decoction (2 points)

【Drug Composition, Dosage and Decoction Method】(3 points)

Mulberry Bark 15g, Scutellaria 12g, Anemarrhena 15g, Gardenia 12g

Bitter Apricot Kernel 9g, Platycodon 9g, Fritillaria 9g, Trichosanthes 15g

Clam Shell (decoct first) 6g, Bamboo Sap 12g, Licorice 3g

3 doses, decocted in water, one dose daily, taken morning and evening.

#### **Case 5**

【Case Summary】

Patient Wu, female, 73 years old, retired. Hospitalized February 12, 2019.

Patient has had recurrent gingival bleeding and cutaneous petechiae and ecchymoses since 2016. Three days ago discovered red urine, obvious gingival bleeding, with dizziness and fatigue, lumbar soreness and tinnitus, dry mouth, tidal fever with night sweats, poor sleep. No headache, no cough with blood-streaked sputum, no chest tightness or palpitation, no nausea and vomiting, no abdominal pain, diarrhea, or tarry stools, no urinary frequency, urgency, or pain, no lower limb edema, came for consultation. Alert consciousness, scattered bleeding points, purpura, and ecchymoses on limbs, densely distributed on bilateral lower limbs. Red tongue with scanty fluid, thin coating, fine rapid pulse.

【Answer Requirements】

(1) Based on the above case summary, please provide: TCM disease diagnosis, TCM syndrome diagnosis, TCM diagnostic basis (including etiology and pathogenesis analysis), TCM treatment principles, formula, drug composition, dosage and decoction method.

(2) TCM disease differentiation: Please differentiate from erysipelas.

【TCM Disease Diagnosis】Blood syndrome - purpura (3 points)

【TCM Syndrome Diagnosis】Yin deficiency fire hyperactivity syndrome (3 points)

【TCM Diagnostic Basis】

The patient presents with "cutaneous petechiae and ecchymoses, gingival bleeding" as main symptoms, thus can be diagnosed as blood syndrome purpura (1 point); based on "cutaneous petechiae and ecchymoses, red urine, obvious gingival bleeding, with dizziness and fatigue, lumbar soreness and tinnitus, dry mouth, tidal fever with night sweats, poor sleep, red tongue with scanty fluid, thin coating, fine rapid pulse," it can be diagnosed as yin deficiency fire hyperactivity syndrome (1 point). This is caused by deficient fire internally blazing, scorching vessels, blood overflowing into skin (2 points).

【TCM Disease Differentiation】

Erysipelas belongs to TCM external dermatological disease, named for skin color red like cinnabar, mild cases fade on pressure, severe cases do not fade on pressure, with local skin burning hot swelling and pain, different from purpura. (3 points)

【TCM Treatment Principle】Nourish yin and descend fire, calm collaterals and stop bleeding (2 points)

【Formula Name】Modified Qiangen Powder (2 points)

【Drug Composition, Dosage and Decoction Method】(3 points)

Madder Root 15g, Scutellaria 9g, Donkey-hide Gelatin (dissolve) 9g, Arborvitae Leaf 15g

Raw Rehmannia 15g, Moutan 15g, Ligustrum 15g, Eclipta 15g

Scrophularia 9g, Licorice 6g, Cornus 12g, Chinese Yam 15g

3 doses, decocted in water. One dose daily, taken morning and evening.

#### **Case 6**

【Case Summary】

Patient Jiang, female, 52 years old, teacher. Initial consultation June 3, 2019.

Patient has 15-year history of hepatitis, recurrent yellow eyes and body, usually irregular TCM and Western medicine, specifics unknown. One month ago after fatigue again developed yellow eyes and body, dull yellow color, with fatigue, poor appetite, bland tasteless mouth without thirst, aversion to cold, loose stools, epigastric stuffiness and abdominal distension, came for consultation. Pale tongue with greasy coating, soggy slow pulse.

【Answer Requirements】

(1) Based on the above case summary, please provide: TCM disease diagnosis, TCM syndrome diagnosis, TCM diagnostic basis (including etiology and pathogenesis analysis), TCM treatment principles, formula, drug composition, dosage and decoction method.

(2) TCM disease differentiation: Please differentiate from yang jaundice.

【TCM Disease Diagnosis】Jaundice (3 points)

【TCM Syndrome Diagnosis】Yin jaundice - cold-dampness obstruction syndrome (3 points)

【TCM Diagnostic Basis】

The patient presents with "yellow eyes and body" as main symptoms, thus can be diagnosed as jaundice (1 point); based on "dull yellow color, with fatigue, poor appetite, bland tasteless mouth without thirst, aversion to cold, loose stools, epigastric stuffiness and abdominal distension, pale tongue with greasy coating, soggy slow pulse," it can be diagnosed as yin jaundice cold-dampness obstruction syndrome (1 point). This is caused by middle yang not vibrant, cold-dampness staying, liver-gallbladder losing in dredging and draining (2 points).

【TCM Disease Differentiation】

Yang jaundice is mainly dampness-heat, yin jaundice is mainly cold-dampness. Yang jaundice belongs to heat syndrome, excess syndrome, bright yellow color like orange peel; acute onset, short course, often accompanied by fever, dry bitter mouth, yellow greasy tongue coating, soggy rapid pulse. Yin jaundice belongs to cold syndrome, deficiency syndrome, dull yellow color, long course, slow disease progression, often accompanied by aversion to cold and mental fatigue, abdominal distension, loose stools, white greasy tongue coating, pale tongue, deep slow pulse. (3 points)

【TCM Treatment Principle】Warm middle and transform dampness, strengthen spleen and harmonize stomach (2 points)

【Formula Name】Modified Yinchen Shufu Decoction (2 points)

【Drug Composition, Dosage and Decoction Method】(3 points)

Artemisia capillaris 15g, Aconite (decoct first) 3g, White Atractylodes 10g, Dried Ginger 6g

Poria 12g, Curcuma 10g, Magnolia 6g, Licorice 6g

3 doses, decocted in water. One dose daily, taken morning and evening.

#### **Case 7**

【Case Summary】

Patient Li, female, 29 years old, civil servant, married. Consultation June 7, 2017.

Patient has had choking cough and shortness of breath for nearly one month, scanty sticky sputum, occasional hemoptysis with fresh red blood, progressive weight loss, afternoon tidal fever, five-center vexing heat, night sweats, came for consultation. Red tongue, thin yellow peeling coating, fine rapid pulse. Her spouse was diagnosed with pulmonary tuberculosis half year ago.

【Answer Requirements】

(1) Based on the above case summary, please provide: TCM disease diagnosis, TCM syndrome diagnosis, TCM diagnostic basis (including etiology and pathogenesis analysis), TCM treatment principles, formula, drug composition, dosage and decoction method.

(2) TCM disease differentiation: Please differentiate from lung atrophy.

【TCM Disease Diagnosis】Lung consumption (3 points)

【TCM Syndrome Diagnosis】Deficient fire scorching lung syndrome (3 points)

【TCM Diagnostic Basis】

Patient's spouse is pulmonary tuberculosis patient, patient presents with "choking cough and shortness of breath, hemoptysis, tidal fever, night sweats, progressive weight loss" as main symptoms, thus can be diagnosed as lung consumption (1 point); based on "occasional hemoptysis with fresh red blood, afternoon tidal fever, five-center vexing heat, irritability and anger, night sweats, red tongue, thin yellow peeling coating, fine rapid pulse," it can be diagnosed as deficient fire scorching lung syndrome (1 point). This is caused by lung-kidney yin injury, water deficient fire exuberant, dryness-heat internally scorching, collateral damage blood overflow (2 points).

【TCM Disease Differentiation】

Both lung consumption and lung atrophy are chronic consumptive diseases located in lung, but lung atrophy is late-stage transformation of various chronic lung diseases, such as lung abscess, lung consumption, chronic cough leading to lung leaf atrophy and disuse, all can become atrophy. Late-stage lung consumption can also transform to lung atrophy. But must clarify lung consumption does not equal lung atrophy, they have causal and severity differences. If late-stage lung consumption presents with dry cough, expectoration of turbid saliva, it has transformed to lung atrophy condition. Clinically lung atrophy presents mainly with cough and expectoration of turbid saliva as main symptom, while lung consumption is characterized by cough, hemoptysis, tidal fever, night sweats. (3 points)

【TCM Treatment Principle】Nourish yin and descend fire (2 points)

【Formula Name】Baihe Gujin Decoction combined with Qinjiao Biejia Powder modified (2 points)

【Drug Composition, Dosage and Decoction Method】(3 points)

Lily Bulb 10g, Stemona 15g, South Adenophora 15g, North Adenophora 15g

Polygonatum odoratum 10g, Bletilla 10g, Moutan 10g, Raw Rehmannia 15g

Ophiopogon 10g, Gentiana macrophylla 10g, Turtle Shell (decoct first) 30g

3 doses, decocted in water. One dose daily, taken morning and evening.

#### **Case 8**

【Case Summary】

Patient Zheng, male, 58 years old, married, cadre. Initial consultation May 5, 2018.

Patient has 10-year smoking history, 3-year elevated blood lipid history. For half year often during fast walking or carrying heavy loads upstairs experiences substernal oppressive pain, severe cases radiating to neck or left shoulder, relieved by stopping walking, usually lasting about 5 minutes. Intermittently taking Danshen tablets, Musk Heart Pills, symptoms vary in severity. Current symptoms: hidden chest pain, intermittent, with palpitation and shortness of breath, lassitude and fatigue, low weak voice, easy sweating with movement. Fat tongue body, pale red quality, teeth marks on border, thin white coating, deficient fine pulse.

【Answer Requirements】

(1) Based on the above case summary, please provide: TCM disease diagnosis, TCM syndrome diagnosis, TCM diagnostic basis (including etiology and pathogenesis analysis), TCM treatment principles, formula, drug composition, dosage and decoction method.

(2) TCM disease differentiation: Please differentiate from gastric pain.

【TCM Disease Diagnosis】Chest impediment (3 points)

【TCM Syndrome Diagnosis】Qi-yin deficiency syndrome (3 points)

【TCM Diagnostic Basis】

The patient presents with "substernal oppressive pain" as main symptom, thus can be diagnosed as chest impediment (1 point); based on "hidden chest pain, intermittent, with palpitation and shortness of breath, lassitude and fatigue, low weak voice, easy sweating with movement, fat tongue body, pale red quality, teeth marks on border, thin white coating, deficient fine pulse," it can be diagnosed as qi-yin deficiency syndrome (1 point). This is caused by heart qi insufficiency, yin-blood deficiency, blood flow stasis (2 points).

【TCM Disease Differentiation】

Heart is above epigastrium, epigastrium is below heart, so there is gastric epigastrium corresponding to heart pain description, due to close location. Atypical chest impediment pain can be in gastric epigastrium area, easily confused. But chest impediment presents mainly with oppressive pain, very short duration, although related to diet, rest and medication often can relieve. Gastric pain is related to diet, presents mainly with distending pain, local tenderness, longer duration, often accompanied by acid regurgitation, noisy stomach, belching, hiccup and other gastric symptoms. (3 points)

【TCM Treatment Principle】Benefit qi and nourish yin, activate blood and unblock vessels (2 points)

【Formula Name】Shengmai Powder combined with Renshen Yangying Decoction modified (2 points)

【Drug Composition, Dosage and Decoction Method】(3 points)

Ophiopogon 10g, Schisandra 9g, Codonopsis 15g, Poria 10g

White Atractylodes 10g, Licorice 6g, Angelica 12g, Prepared Rehmannia 15g

Peony 10g, Tangerine Peel 12g, Polygala 10g

3 doses, decocted in water. One dose daily, taken morning and evening.

#### **Case 9**

【Case Summary】

Patient Lu, male, 48 years old, office worker. Hospitalized January 28, 2021. Has drinking and smoking history.

Patient has 3-year hypertension history, usually takes Zhenju Jiangya tablets twice daily, one tablet each time, reports good blood pressure control. Yesterday afternoon suddenly felt left limb numbness and weakness, with headache, dizziness, bitter mouth and dry throat, nausea, no improvement after rest. Next day felt left limb weakness worsened and developed unclear speech, came to our hospital. Throughout disease process remained conscious, no vomiting, limb convulsions, or incontinence. Alert consciousness, flushed face and red ears, slightly coarse breath sounds in both lungs, HR 98 beats/min, regular rhythm, soft abdomen without tenderness or rebound tenderness, no bilateral lower limb edema. Red tongue, yellow greasy coating, wiry forceful pulse.

【Answer Requirements】

(1) Based on the above case summary, please provide: TCM disease diagnosis, TCM syndrome diagnosis, TCM diagnostic basis (including etiology and pathogenesis analysis), TCM treatment principles, formula, drug composition, dosage and decoction method.

(2) TCM disease differentiation: Please differentiate from atrophy syndrome.

【TCM Disease Diagnosis】Stroke (meridian involvement) (3 points)

【TCM Syndrome Diagnosis】Wind-yang ascending disturbance syndrome (3 points)

【TCM Diagnostic Basis】

The patient presents with "sudden left limb numbness and weakness, with headache, dizziness, unclear speech" as main symptoms, thus can be diagnosed as stroke meridian involvement (1 point); based on "left limb weakness, unclear speech, alert consciousness, flushed face and red ears, soft abdomen without tenderness or rebound tenderness, no bilateral lower limb edema, red tongue, yellow greasy coating, wiry forceful pulse," it can be diagnosed as wind-yang ascending disturbance syndrome (1 point). This is caused by liver fire hyperactivity, yang hyperactivity transforming wind, transversely running vessel collaterals (2 points).

【TCM Disease Differentiation】

Atrophy syndrome can have limb paralysis, movement weakness similar to stroke manifestations; stroke late-stage hemiplegia unable to recover can also show muscle wasting, sinew laxity, both should be differentiated. But atrophy syndrome generally has slow onset, with bilateral lower limb paralysis or quadriplegia, or muscle atrophy, sinew twitching muscle tremor more common; while stroke limb paralysis mostly has acute sudden onset, and mainly hemiplegia. Atrophy syndrome has no spirit clouding at onset, stroke often has different degrees of spirit clouding. (3 points)

【TCM Treatment Principle】Clear liver and drain fire, calm wind and subdue yang (2 points)

【Formula Name】Modified Tianma Gouteng Drink (2 points)

【Drug Composition, Dosage and Decoction Method】(3 points)

Gastrodia 10g, Uncaria (add later) 15g, Abalone Shell (decoct first) 30g, Prunella 30g

Scutellaria 10g, Achyranthes 15g, Gardenia 10g, Chrysanthemum 10g

3 doses, decocted in water. One dose daily, taken morning and evening.

#### **Case 10**

【Case Summary】

Patient Huang, male, 23 years old, unmarried. Initial consultation March 30, 2020.

Patient had left head trauma in childhood, usually has dizziness and headache, episodic right limb convulsions for 2 years, never regular treatment. Current symptoms: episodic right limb convulsions lasting about 5 minutes, no obvious discomfort between episodes, with left headache, blue-purple face and lips, dark red tongue with stasis spots, thin white coating, wiry pulse.

【Answer Requirements】

(1) Based on the above case summary, please provide: TCM disease diagnosis, TCM syndrome diagnosis, TCM diagnostic basis (including etiology and pathogenesis analysis), TCM treatment principles, formula, drug composition, dosage and decoction method.

(2) TCM disease differentiation: Please differentiate from stroke.

【TCM Disease Diagnosis】Epilepsy (3 points)

【TCM Syndrome Diagnosis】Remission period - stasis obstructing brain collaterals syndrome (3 points)

【TCM Diagnostic Basis】

The patient presents with "episodic right limb convulsions lasting about 5 minutes, no obvious discomfort between episodes" as main symptoms, thus can be diagnosed as epilepsy (1 point); based on "with left headache, blue-purple face and lips, dark red tongue with stasis spots, thin white coating, wiry pulse," it can be diagnosed as remission period stasis obstructing brain collaterals syndrome (1 point). This is caused by blood stasis obstructing orifices, brain collaterals blocked, brain spirit losing nourishment and wind movement (2 points).

【TCM Disease Differentiation】

Typical epileptic attack and stroke both have sudden collapse, unconsciousness, but epilepsy has recurrent attack history, during attack has foaming at mouth, upward-gazing eyes, limb convulsions, or strange cries, can spontaneously recover, no hemiplegia, mouth-tongue deviation symptoms, while stroke falls silently, long-lasting coma, often has hemiplegia and other sequelae after awakening. (3 points)

【TCM Treatment Principle】Activate blood and transform stasis, calm wind and unblock collaterals (2 points)

【Formula Name】Modified Tongqiao Huoxue Decoction (2 points)

【Drug Composition, Dosage and Decoction Method】(3 points)

Red Peony 10g, Chuanxiong 9g, Peach Kernel 9g

Safflower 9g, Musk 6g, Old Scallion 10g

Earthworm 10g, Bombyx Batryticatus 10g, Scorpion 10g

3 doses, decocted in water. One dose daily, taken morning and evening.

#### **Case 11**

【Case Summary】

Patient Wang, female, 62 years old, married, farmer. Initial consultation February 9.

Patient for nearly 20 years, every time catching cold has recurrent dyspnea and cough, symptoms gradually worsen yearly, multiple hospitalizations. Recent 2 months after weather turned cold, dyspnea and cough recurred. Main symptoms: dyspnea with chest tightness, exhale more inhale less, dyspnea especially severe with movement, qi unable to continue, thin weak appearance, sweating with cold limbs, blue-green face with purple lips, pale tongue with white coating, minute fine pulse.

【Answer Requirements】

(1) Based on the above case summary, please provide: TCM disease diagnosis, TCM syndrome diagnosis, TCM diagnostic basis (including etiology and pathogenesis analysis), TCM treatment principles, formula, drug composition, dosage and decoction method.

(2) TCM disease differentiation: Please differentiate from asthma disease.

【TCM Disease Diagnosis】Dyspnea syndrome (3 points)

【TCM Syndrome Diagnosis】Kidney deficiency unable to absorb syndrome (3 points)

【TCM Diagnostic Basis】

The patient presents with "dyspnea with chest tightness" as main symptom, thus can be diagnosed as dyspnea syndrome (1 point); based on "exhale more inhale less, dyspnea with movement, qi unable to continue, thin weak appearance, sweating with cold limbs, blue-green face with purple lips, pale tongue with white coating, minute fine pulse," it can be diagnosed as deficiency dyspnea kidney deficiency unable to absorb syndrome (1 point). This is caused by lung disease affecting kidney, lung-kidney both deficient, qi losing absorption (2 points).

【TCM Disease Differentiation】

Both dyspnea syndrome and asthma disease have rapid breathing and difficulty manifestations. Dyspnea refers to breathing, as breathing difficulty, even opening mouth lifting shoulders, shaking body holding belly, is a symptom of various acute and chronic lung system diseases; asthma refers to sound, must have wheezing sound in throat, also with breathing difficulty, is a repeatedly attacking independent disease. Dyspnea not necessarily includes asthma, while asthma must include dyspnea. (3 points)

【TCM Treatment Principle】Supplement kidney and absorb qi (2 points)

【Formula Name】Jingui Shenqi Pill combined with Shenha Powder modified (2 points)

【Drug Composition, Dosage and Decoction Method】(3 points)

Prepared Aconite (decoct first) 6g, Prepared Rehmannia 15g, Alisma 15g, Chinese Yam 15g

Gecko Powder (wrapped) 3g, Cornus 12g, Moutan 9g, Poria 12g

Cinnamon (add later) 6g, Walnut Meat 12g, Ginseng 9g, Schisandra 9g

3 doses, decocted in water. One dose daily, taken morning and evening.

#### **Case 12**

【Case Summary】

Patient, female, 28 years old. Initial consultation October 21, 2021.

Patient usually irritable and easily angered, flushed face with red eyes, vexation insomnia, unsatisfactory expectoration, bitter mouth and dry throat, constipation with yellow urine, sleepless all night, red eyes, during attacks has collapse convulsions, foaming, roaring. Red tongue, yellow greasy coating, wiry slippery rapid pulse.

【Answer Requirements】

(1) Based on the above case summary, please provide: TCM disease diagnosis, TCM syndrome diagnosis, TCM diagnostic basis (including etiology and pathogenesis analysis), TCM treatment principles, formula, drug composition, dosage and decoction method.

(2) TCM disease differentiation: Please differentiate from syncope syndrome.

【TCM Disease Diagnosis】Epilepsy (3 points)

【TCM Syndrome Diagnosis】Remission period - liver fire phlegm-heat syndrome (3 points)

【TCM Diagnostic Basis】

The patient presents with "collapse convulsions, foaming, roaring" as main symptoms, thus can be diagnosed as epilepsy (1 point); based on "usually irritable and easily angered, vexation insomnia, flushed face with red eyes, unsatisfactory expectoration, bitter mouth and dry throat, constipation with yellow urine, sleepless all night, red eyes, red tongue, yellow greasy coating, wiry slippery rapid pulse," it can be diagnosed as phlegm-fire disturbing spirit syndrome (1 point). This is caused by liver constraint transforming fire, phlegm-fire internally exuberant, ascending to disturb original spirit (2 points).

【TCM Disease Differentiation】

Besides sudden collapse and unconsciousness main symptoms, syncope syndrome also has pale complexion, cold limbs, or mouth lockjaw, clenched fists, finger urgency, without foaming at mouth, upward-gazing eyes, limb convulsions and strange crying symptoms, clinically not difficult to differentiate. (3 points)

【TCM Treatment Principle】Clear liver and drain fire, transform phlegm and calm heart (2 points)

【Formula Name】Longdan Xiegan Decoction combined with Ditan Decoction modified (2 points)

【Drug Composition, Dosage and Decoction Method】(3 points)

Gentian 9g, Indigo 10g, Aloe 12g, Rhubarb 9g

Scutellaria 10g, Gardenia 9g, Ginger Pinellia 10g, Arisaema with Bile 12g

Aucklandia 12g, Bitter Orange 9g, Poria 12g, Red Tangerine Peel 10g

Ginseng 12g, Acorus 9g, Musk 5g

3 doses, decocted in water. One dose daily, taken morning and evening.

#### **Case 13**

【Case Summary】

Patient, male, 78 years old, married. Consultation December 2019.

Patient has 12-year hypertension history, 3 years of head shaking and limb tremor. Symptoms: head shaking and limb tremor, unable to hold bowls and chopsticks, lumbar and knee soreness, dizziness, tinnitus, forgetfulness, red tongue, red crimson tongue without coating, fine rapid pulse.

【Answer Requirements】

(1) Based on the above case summary, please provide: TCM disease diagnosis, TCM syndrome diagnosis, TCM diagnostic basis (including etiology and pathogenesis analysis), TCM treatment principles, formula, drug composition, dosage and decoction method.

(2) TCM disease differentiation: Please differentiate from convulsions.

【TCM Disease Diagnosis】Tremor syndrome (3 points)

【TCM Syndrome Diagnosis】Marrow sea insufficiency syndrome (3 points)

【TCM Diagnostic Basis】

The patient presents with "head shaking and limb tremor, unable to hold bowls and chopsticks" as main symptoms, thus can be diagnosed as tremor syndrome (1 point); based on "lumbar and knee soreness, dizziness, tinnitus, forgetfulness, red tongue, red crimson tongue without coating, fine rapid pulse," it can be diagnosed as marrow sea insufficiency syndrome (1 point). This is caused by marrow sea insufficiency, spirit mechanism losing nourishment, limb sinew vessels losing control (2 points).

【TCM Disease Differentiation】

Convulsions are spasms, mostly seen in acute febrile diseases or certain chronic disease acute attacks, convulsions mostly continuous with sometimes brief intermittent periods, hands and feet flexion-extension pulling, relaxation alternating, some patients can have fever, upward-gazing eyes, spirit clouding symptoms; tremor syndrome is a chronic disease process, with head-neck, hands-feet involuntary tremor and shaking as main symptoms, hands-feet tremor movements have small amplitude, high frequency, without limb convulsion pulling and fever, spirit clouding symptoms, combined with medical history analysis, both not difficult to differentiate. (3 points)

【TCM Treatment Principle】Fill essence and supplement marrow, nourish yin and calm wind (2 points)

【Formula Name】Guiluke Erxian Paste combined with Dading Fengzhu modified (2 points)

【Drug Composition, Dosage and Decoction Method】(3 points)

Turtle Shell (decoct first) 12g, Turtle Shell (decoct first) 10g, Uncaria 6g, Egg Yolk 3g

Raw Oyster Shell (decoct first) 10g, Donkey-hide Gelatin (dissolve) 6g, Lycium 9g, White Peony 9g

Deer Antler Glue (dissolve) 3g, Schisandra 9g, Prepared Rehmannia 9g, Raw Rehmannia 10g

Ophiopogon 9g, Cannabis Seed 6g, Ginseng 6g, Chinese Yam 9g

Poria 9g, Licorice 6g

3 doses, decocted in water. One dose daily, taken morning and evening.

#### **Case 14**

【Case Summary】

Patient, male, 71 years old, retired. Consultation December 2019.

Patient has recurrent cough and dyspnea for over 20 years, condition worsened in recent 3 years, requires hospitalization twice yearly. Patient just discharged 2 weeks ago, yesterday after fatigue, chest distension and fullness, shortness of breath with dyspnea, cough with profuse foamy sputum, epigastric focal distension with poor appetite, lassitude and fatigue, dark tongue, thin greasy coating, small slippery pulse.

【Answer Requirements】

(1) Based on the above case summary, please provide: TCM disease diagnosis, TCM syndrome diagnosis, TCM diagnostic basis (including etiology and pathogenesis analysis), TCM treatment principles, formula, drug composition, dosage and decoction method.

(2) TCM disease differentiation: Please differentiate from asthma disease.

【TCM Disease Diagnosis】Lung distension (3 points)

【TCM Syndrome Diagnosis】Phlegm turbidity blocking lung syndrome (3 points)

【TCM Diagnostic Basis】

Patient has recurrent cough and dyspnea for over 20 years, this time presents with "chest distension and fullness, shortness of breath with dyspnea, cough with profuse sputum" as main symptoms, thus can be diagnosed as lung distension (1 point); based on "chest distension and fullness, shortness of breath with dyspnea, cough with profuse foamy sputum, epigastric focal distension with poor appetite, lassitude and fatigue, dark tongue, thin greasy coating, small slippery pulse," it can be diagnosed as phlegm turbidity blocking lung syndrome (1 point). This is caused by lung deficiency spleen weakness, phlegm turbidity internally accumulating, lung losing diffusion and descent (2 points).

【TCM Disease Differentiation】

Both lung distension and asthma disease present with cough and ascending qi, dyspnea and fullness as main symptoms, have similarities. Difference is lung distension is gradual development from various chronic lung system diseases, besides cough and dyspnea, also has chest distension and fullness, palpitation, purple lips and nails, abdominal distension with limb swelling symptoms; asthma disease is repeatedly attacking disease, characterized by wheezing sound in throat. From the mutual relationship of dyspnea syndrome and asthma disease, lung distension can belong to dyspnea syndrome category, chronic dyspnea can also develop into lung distension. (3 points)

【TCM Treatment Principle】Transform phlegm and descend qi, strengthen spleen and benefit lung (2 points)

【Formula Name】Suzi Jiangqi Decoction combined with Sanzi Yangqin Decoction (2 points)

【Drug Composition, Dosage and Decoction Method】(3 points)

Perilla Seed 9g, White Mustard Seed 9g, Pinellia 9g, Peucedanum 6g

Radish Seed 9g, Angelica 6g, Magnolia 6g, Tangerine Peel 6g

White Atractylodes 9g, Poria 9g, Licorice 6g

3 doses, decocted in water. One dose daily, taken morning and evening.

#### **Case 15**

【Case Summary】

Patient Xu, male, 47 years old. Consultation November 20, 2020.

Chief complaint: Hematemesis once.

History: Patient has 11-year gastric disease history, every autumn-winter season often attacks. Recent 2 years had 3 episodes of hematemesis and melena, previously underwent fiberoptic gastroscopy at city hospital, diagnosed as "gastric ulcer." Day before consultation drank heavily, then gastric pain gradually worsened, self-administered famotidine with slight pain relief, next morning felt chest tightness, nausea, then vomited coffee-colored fluid about 150mL with food residue inside. Family immediately sent to hospital. Current symptoms: epigastric distension and fullness with stuffiness, halitosis, black formed stool. Red tongue, yellow coating, rapid pulse. Examination: blood pressure 130/80mmHg, stool occult blood ++++.

【Answer Requirements】

(1) Based on the above case summary, please provide: TCM disease diagnosis, TCM syndrome diagnosis, TCM diagnostic basis (including etiology and pathogenesis analysis), TCM treatment principles, formula, drug composition, dosage and decoction method.

(2) TCM disease differentiation: Please differentiate from hemoptysis.

【TCM Disease Diagnosis】Blood syndrome - hematemesis (3 points)

【TCM Syndrome Diagnosis】Stomach heat abundant syndrome (3 points)

【TCM Diagnostic Basis】

The patient presents with "vomiting coffee-colored fluid with food residue inside" as main symptom, thus can be diagnosed as blood syndrome hematemesis (1 point); based on "epigastric distension and fullness with stuffiness, halitosis, black formed stool, red tongue, yellow coating, rapid pulse," it can be diagnosed as stomach heat abundant syndrome (1 point). This is caused by stomach heat internal constraint, heat injuring stomach collaterals (2 points).

【TCM Disease Differentiation】

Both blood types exit through mouth, but completely different. Hemoptysis is blood from lung, through airways with cough, blood color mostly fresh red, often mixed with sputum, before hemoptysis mostly has cough, chest tightness, throat itch symptoms, after massive hemoptysis, can see blood-streaked sputum for days, stool generally not black. Hematemesis is blood from stomach, through vomiting, blood color purple-dark, often mixed with food residue, before hematemesis mostly has gastric discomfort or gastric pain, nausea symptoms, after hematemesis no blood-streaked sputum, but stool mostly black. (3 points)

【TCM Treatment Principle】Clear stomach and drain fire, transform stasis and stop bleeding (2 points)

【Formula Name】Xiexin Decoction combined with Shihui Powder modified (2 points)

【Drug Composition, Dosage and Decoction Method】(3 points)

Scutellaria 10g, Coptis 6g, Madder Root 15g, Arborvitae Leaf 15g

Rhubarb 10g, Gardenia 10g, Hematite (decoct first) 15g, Bamboo Shavings 10g

Greater Thistle 15g, Moutan 10g, Notoginseng Powder (dissolve) 3g, Licorice 6g

3 doses, decocted in water. One dose daily, taken morning and evening.
