# Supplementary material for: Digitally Assisted Clinical Decision-Making in Traditional Chinese Medicine: Comparative Study of 5 Large Language Models
Source: JMIR Form Res. 2026 Mar 2;10:e80167. doi: 10.2196/80167 (PMC12954686; doi:10.2196/80167)
Supplement: Multimedia Appendix 3 [file formative-v10-e80167-s003.doc]

## Multimedia Appendix 3: Complete Materials of 5 Clinical Cases

### Case 1[1]

Name: - Gender: Female Age: 82 years Consultation date: February 16, 2020

Chief complaint: Confirmed COVID-19 for over 5 days

Present illness: From January 23, 2020, had meals with daughter-in-law (confirmed COVID-19 patient on February 5) for 5 consecutive days. Throat swab tested positive on February 12, subsequently admitted to Kaijiang County Hospital. During expert consultation, patient's blood oxygen saturation dropped to 80% without chest tightness or dyspnea, transferred to Dazhou Central Hospital on February 16. Current symptoms: cough, wheezing, chest tightness, fatigue with poor appetite, no bowel movement for 2 days.

Physical examination: Temperature 36.8°C, coarse breath sounds in both lungs.

Specialized examination: Chest CT shows bilateral diffuse multiple patchy infiltrates, more prominent in bilateral lower lungs. SpO2 84%, nasal oxygen SpO2 93%, mask oxygen SpO2 96%.

Tongue and coating: Dark red tongue with thick, dry yellow coating.

Laboratory tests: WBC 1.77×10^9/L, neutrophil percentage 66.7%, lymphocytes 0.34×10^9/L, lymphocyte percentage 19.2%; CRP 8.6mg/L, procalcitonin 0.045ng/mL; blood gas analysis: pH 7.45, PCO2 36.8mmHg, PO2 88mmHg.

Based on the above case, please provide Western medicine disease diagnosis, TCM disease diagnosis, TCM syndrome diagnosis, TCM treatment principles, formula, drug composition, and precautions.

### Case 2[2]

Name: Xu XX Gender: Female Age: 46 years Consultation date: January 5, 2006

Chief complaint: Recurrent small ulcers in oral cavity and vulva

Present illness: Patient began experiencing recurrent small ulcers in oral cavity and vulva in 2001, particularly severe before and during menstruation, showing cyclical patterns. Diagnosed as "Behçet's disease" at a Shanghai hospital. Continuous hormone treatment for 2 years was ineffective, and Tripterygium wilfordii caused amenorrhea. During this period, hundreds of Chinese medicine prescriptions were tried, including Huanglian Jiedu Decoction, Rhinoceros Horn and Rehmannia Decoction, Longdan Xiegan Decoction, without significant effect. Current symptoms: oral ulcers healed. Multiple small ulcers on labial mucosa, ulcer surface dark red with burning pain. Irritability, red complexion, constipation with reddish urine.

Physical examination: Multiple small ulcers on labial mucosa, ulcer surface dark red with burning pain.

Tongue and coating: Red tongue with scanty coating

Pulse: Fine and rapid

Based on the above case, please provide Western medicine disease diagnosis, TCM disease diagnosis, TCM syndrome diagnosis, TCM treatment principles, formula, drug composition, and precautions.

### Case 3[3]

Name: - Gender: Male Age: 55 years Consultation date: April 10, 2017

Chief complaint: Limb tremor and unsteady gait for 2 years, jaw tremor for 3 months

Present illness: 2 years ago, due to paroxysmal limb tremor and unsteady gait, diagnosed as multiple system atrophy at a tertiary hospital neurology department, treated with levodopa/carbidopa 0.125g, twice daily, condition did not progress further, usually had paroxysmal limb tremor, but no head tremor or shaking. 3 months ago, after argument with family, developed jaw tremor, unclear speech, slow movements. Local hospital brain MRI showed no obvious infarction, so no medication was added, but symptoms did not improve. Paroxysmal jaw tremor, episodes lasting 15 minutes to 1 hour, affecting speech and eating. Usually has dizziness, mental fatigue, occasional unprovoked anger. Current symptoms: jaw tremor involving corners of mouth, body stiffness and rigidity, dull expression when not questioned, excited expression when questioned, unclear speech, slightly fast speech rate, incomplete sentences, unclear pronunciation. Feels generalized discomfort, limbs also occasionally tremor uncontrollably, unsteady gait, heavy head and light feet, cotton-stepping sensation, restless sleep with easy awakening and many dreams, dry mouth and bitter taste, normal appetite, constipation requiring enema assistance every 2-3 days.

Past history: Hypertension for 3 years

Physical examination: Jaw tremor involving corners of mouth, body stiffness and rigidity, dull expression when not questioned, excited expression when questioned, unclear speech, slightly fast speech rate, incomplete sentences, unclear pronunciation.

Tongue and coating: Red tongue with thin yellow coating

Pulse: Wiry and rapid

Laboratory tests: Brain MRI: mild white matter degeneration, cerebellar atrophy, with slight inflammation in right ethmoid sinus. Carotid ultrasound: bilateral carotid intima-media thickening. Abdominal ultrasound: bilateral renal cysts. Echocardiogram: left ventricular diastolic dysfunction.

Based on the above case, please provide Western medicine disease diagnosis, TCM disease diagnosis, TCM syndrome diagnosis, TCM treatment principles, formula, drug composition, and precautions.

### Case 4[4]

Name: - Gender: Male Age: 7 years Consultation date: May 6, 2009

Chief complaint: Cutaneous purpura for half year

Present illness: Bilateral lower limb cutaneous purpura, scattered distribution, dull color, appearing and disappearing intermittently, recurring continuously, five-center heat, tidal fever with night sweats, thirst, normal appetite, dry stool with yellow urine.

Physical examination: Dark circles around eyes like "panda eyes," bilateral lower limb cutaneous petechiae and ecchymoses, scattered distribution, dull color, non-blanching on pressure. Dark red throat, otherwise normal.

Tongue and coating: Dark red dry tongue, tortuous sublingual veins, thin scanty coating

Pulse: Fine, rapid and rough

Laboratory tests: Blood routine: normal. Urinalysis: negative.

Based on the above case, please provide Western medicine disease diagnosis, TCM disease diagnosis, TCM syndrome diagnosis, TCM treatment principles, formula, drug composition, and precautions.

### Case 5[5]

Name: - Gender: Female Age: 77 years Consultation date: December 17, 2019

Chief complaint: Dry mouth for 15 years, cutaneous ecchymoses for 6 years

Present illness: Patient developed dry mouth without obvious cause 15 years ago, drinking >2000mL water daily, with dental caries. 6 years ago developed cutaneous petechiae and ecchymoses without obvious cause. External hospital consultation found antinuclear antibody (ANA) 1:320, anti-SSA antibody positive, platelet (PLT) reduction (specific details unknown), ophthalmology consultation indicated dry eye syndrome, salivary gland scintigraphy showed bilateral parotid gland uptake function mildly decreased, considered as SS, treated with methylprednisolone 24mg daily orally, cutaneous ecchymoses resolved, PLT returned to normal range, steroids gradually reduced, then long-term monthly methylprednisolone 2mg treatment. Patient recently found cutaneous ecchymoses again, local hospital blood routine on December 17, 2019: white blood cell (WBC) 2.6×10^9/L, hemoglobin (HGB) 94 g/L, PLT 20×10^9/L, came for consultation. Current symptoms: patient has recurrent epistaxis, worse with fatigue, with dizziness, mental fatigue, shortness of breath and laziness to speak, sweating with movement, sallow complexion, poor appetite with loose stool, abdominal distension after eating, pale lip color, mild bilateral lower limb edema.

Past history: Lumbar compression fracture 5 years ago

Physical examination: Temperature: 37.1°C, heart rate: 91 beats/min, respiratory rate: 19 breaths/min, blood pressure: 170/70mmHg (1mmHg≈0.133 kPa). Alert consciousness, poor spirit, sallow complexion, coarse breath sounds in both lungs without obvious dry or wet rales, regular heart rhythm without murmur, abdominal distension without tenderness or rebound tenderness, scattered petechiae and ecchymoses throughout body, mild bilateral lower limb edema.

Tongue and coating: Red tongue with scanty coating

Pulse: Fine

Laboratory tests: Thoracolumbar spine MRI (November 7, 2019): T10, L3 vertebral compression fractures, L1 vertebral compression fracture post-surgery. December 18, 2019 blood routine: WBC 3.4×10^9/L, HGB 89g/L, PLT 22×10^9/L. Antinuclear antibody panel: ANA 1:320 positive, anti-SSA antibody positive, anti-Ro52 antibody positive.

Based on the above case, please provide Western medicine disease diagnosis, TCM disease diagnosis, TCM syndrome diagnosis, TCM treatment principles, formula, drug composition, and precautions.

### References

1. Peng Q, Yu Y, Wen X, et al. Traditional Chinese Medicine treatment of severe COVID-19 patients: typical cases. Chinese Journal of Traditional Chinese Medicine. 2020;35(7):3455-3459.

2. Chen Y, Qian H. Clinical experience in treating perimenopausal related diseases with kidney-tonifying Chinese medicine. Journal of Traditional Chinese Medicine. 2011;52(2):100-101. doi: 10.13288/j.11-2166/r.2011.02.004.

3. Wang P, Wang B. Wang Bangcai's treatment of atrophy syndrome: a case study. Chinese Journal of Traditional Chinese Medicine. 2024;39(11):5921-5923.

4. Chen W, Ding Y, Yan Y, et al. Master Ding Ying's clinical treatment approach for allergic purpura (cutaneous type): insights and analysis. Chinese Journal of Traditional Chinese Medicine. 2024;39(8):4091-4094.

5. He Z, Yao H, Yang K, et al. Fan Yongsheng's academic experience in treating Sjögren's syndrome with thrombocytopenia: an analysis. Chinese Journal of Traditional Chinese Medicine. 2023;38(12):5854-5857.
